# Patient insights into the experience of trying to achieve weight-loss and future expectations upon commencement of a primary care-led weight management intervention: A qualitative, baseline exploration

**DOI:** 10.1371/journal.pone.0270426

**Published:** 2022-06-29

**Authors:** Marie Spreckley, Judith de Lange, Jacob C. Seidell, Jutka Halberstadt

**Affiliations:** Faculty of Science, Department of Health Sciences, Vrije Universiteit Amsterdam, Amsterdam Public Health Research Institute, Amsterdam, Netherlands; Texas A&M University College Station, UNITED STATES

## Abstract

**Introduction:**

The long-term effects of interventions aiming to achieve substantial, sustainable weight loss maintenance have been disappointing. Most people regain their lost weight over time but some seem to be able to maintain their weight loss. We are following the experiences of patients over time prospectively. This study forms the baseline to provide insights into patient experiences prior to entering a primary care-led weight management intervention and their expectations going forward.

**Materials and methods:**

We recruited 21 adult male and female patients of varying ethnicity with a BMI between 27.7kg/m2 and 48.4kg/m2 from a cohort of patients entering a primary care-led weight management intervention. Patients were offered video and audio interview options during the COVID-19 lockdown. In total, twenty chose the audio option, while one chose the video option. The interview format was semi-structured with room for individual exploration.

**Discussion:**

We found that participants experienced feeling unable to control their weight and encountered a multitude of internal and external barriers to weight management. Some had supportive environments, while others experienced discouraging external influences. Though personal characteristics varied, motivations, goals and expected benefits were similar across this cohort. Most participants had previously experienced transient successful weight-loss attempts with varying approaches. COVID-19 was experienced as an opportunity or barrier for change.

**Conclusion:**

This study illustrates the importance of gaining comprehensive insights into the diverse experiences patients encounter when trying to achieve weight loss. Personalized support taking into account individual experiences and circumstances may enhance long-term treatment outcomes. Future research into the complexities of weight management based on individual accounts can aid in the creation of improved treatment protocols.

## Introduction

The global prevalence of overweight and obesity is increasing in developed and developing countries. In 2020, the World Health Organisation (WHO) estimated that overweight and obesity lead to more fatalities than underweight in most areas of the world [1]. This has been driven by a global transformation of food systems, which are continuously growing and evolving to serve a changing global population [2]. The global population is expected to increase from around 7 to around 9 billion by 2050, accompanied by increased purchasing power and urbanisation [3]. Countries are therefore often confronted with the double burden of malnutrition of having communities faced with both under- and overnutrition [1].

The WHO estimates that ca. 1.9 billion adults currently have overweight (ca. 39% of the global population) of which 650 million adults currently have obesity (ca. 13% of the global population) [1]. Having overweight and obesity has been conclusively linked with a variety of weight related co-morbidities, which tend to have longer latency periods, including type 2 diabetes (T2D), hypertension, hypercholesterolemia, sleep apnea, coronary heart disease (CHD), rheumatoid arthritis and osteoarthritis as well as 13 types of cancer [4].

The underlying mechanisms leading to overweight and obesity are comprised of a vast array of often interrelated internal and external mechanisms including biological, medical, social, psychological, economic and infrastructural, amongst others [5]. Research has consistently shown that both weight loss success and adherence can be achieved with varying dietary approaches [6,7]. Behavioural interventions entailing dietary and physical activity protocols have shown to be particularly promising [8–11]. Yet, while short term weight loss attempts using a multitude of methods have shown to be successful at times, weight regain is experienced by the majority of successful dieters [12]. Additionally, patients frequently regain more weight than they initially lost [13]. Notably, however, patients who manage to maintain their weight loss for more than two years also have an increased likelihood of maintaining their weight loss over the following 5 to 10 years [14].

Bariatric surgery currently provides the most successful long-term outcomes, particularly for patients with a BMI over 35kg/m^2^ [15]. However, due to its invasive nature, long-term post-operative adjustments in terms of diet and supplementation as well as potential, severe complications, it is often not a suitable option [15]. Therefore, it is usually recommended to resort to bariatric surgery after a multitude of lifestyle interventions have been attempted without lasting success [15]. Pharmacotherapy is also emerging as a promising alternative to bariatric surgery and addition to behavioural interventions [16].

A further noteworthy area to highlight is the stigma attached to having overweight and obesity. Weight stigma refers to direct and indirect discrimination, stereotyping and exclusion based on an individual’s weight, which can result in a multitude of detrimental social, psychological and physiological consequences [17]. The negative consequences of weight stigma are far reaching and often result in increased weight gain, decreased health and overall quality of life [18,19], regardless of degree of overweight, gender and age [20]. It is also particularly harmful when this results in the hesitation to seek medical advice and support for the above-mentioned weight related co-morbidities. Therefore, studies providing insights into the experiences patients encounter with weight management have the potential to both decrease weight stigma and increase treatment quality by healthcare practitioners (HCPs) working in weight management.

### Aim and objective

The aim of this study is to create a qualitative, baseline exploration of patient experiences with weight management prior to entering a primary care led weight management intervention and to understand their expectations upon treatment commencement. Qualitative research was chosen as it enables the discovery of comprehensive, nuanced insights into individual patient experiences. The primary care setting was chosen as it offered a comprehensive intervention provided by a multidisciplinary team for patients with varying characteristics and circumstances. The primary research question was: What are the characteristics, needs and expectations of patients with a BMI >27kg/m2 who participate in a weight-loss programme upon commencement of the programme? This study is building on the thematic findings of the systematic review previously conducted titled: “Perspectives into the experience of successful, substantial long-term weight-loss maintenance—A Systematic Review” [21].

The objective is to provide insights into the experience of weight management and the resulting external and internal consequences encountered. This will help inform researchers, persons living with obesity and professionals working in obesity management gain a more comprehensive understanding of the experiences patients with overweight and obesity go through during their life time to enable the formulation of suitable treatment protocols. This study also provides the baseline for two currently ongoing studies into the experience of weight management during and after entering a primary care led weight management programme.

## Materials and methods

### Recruitment and participants

The participants in this study were purposefully recruited [22] from a UK based primary care led programme. All 32 potential participants entering the initial pilot programme were contacted via both email and phone and 21 agreed to participate in this research. The 11 contacted participants who did not participate in this study had similar characteristics in terms of age, gender, ethnicity and BMI as the final cohort yet either expressed feeling uncomfortable with being involved or did not respond. Participants were briefed via phone as well as a comprehensive information and consent form prior to the interviews. The interviews were conducted in English and all participants were fluent in English. Since this study was conducted during COVID-19 lockdown in the UK, participants were offered to participate via video or audio call. In total twenty participants chose the audio call option and 1 participant chose the video call option. All participants agreed to be contacted both for the one and two year follow up studies as well, which will explore the experience of having taken part in a weight-loss programme facilitated by a multi-disciplinary clinical team. The final cohort of participants was comprised of 13 female and 8 male participants including 11 with White British ethnicity, 4 with Asian British ethnicity and 6 with African/Caribbean British ethnicity between the ages of 33 and 71 with a BMI between 27.7kg/m^2^ and 48.4kg/m^2^ (Table 1).

**Table 1:** Table of participant characteristics.

P	M/F	Ethnicity	Age	Co-morbidities	Medications / Devices	BMI (kg/m^2^)
P1	female	Caucasian	45	Irritable bowel syndrome (IBS), gallstones, chronic pancreatitis, Gastro-oesophageal reflux disease (GORD)	Omeprazole	42.6
P2	female	Caucasian	34	Infertility, chronic knee pain	None	36.6
P3	Male	Black	45	T2D, Hypertension, hypercholesterolemia	Atorvastatin, amlodipine	30.6
P4	Male	Asian	41	T2D, Hypertension	Metformin, losartan	33.8
P5	female	Caucasian	44	Depression	Prozac	28.7
P6	Male	Black	35	Sleep apnoea	CPAP machine	32.1
P7	Male	Black	34	Severe gastrointestinal (GI) complications	None	31.5
P8	female	Black	61	T2D, Hypertension, Hypercholesterolemia, arthritis	Metformin, atorvastatin, furosemide	40.5
P9	female	Caucasian	66	Colon cancer, hypertension, asthma	Belok-Zok cor, symbicort inhaler	29.1
P10	Male	Caucasian	71	Hypercholesterolemia, asthma, arthritis	Simvastatin, symbicort inhaler	27.7
P11	female	Black	55	Hypertension	None	29
P12	female	Asian	42	T2D, Hypertension	Losartan	29.4
P13	female	Caucasian	33	Depression, anxiety, arthritis	None	43.3
P14	female	Asian	46	Prediabetes, GORD	None	36.1
P15	Male	Black	55	T2D, hypertension, chronic obstructive pulmonary disease (COPD)	Metformin, atorvastatin, doxazosin, losartan, aspirin, amlodipine	48.4
P16	female	Caucasian	41	None	None	36.6
P17	Male	Caucasian	45	Asthma	Symbicort inhaler	39.8
P18	female	Caucasian	68	T2D, hypertension, arthritis, GORD	Metformin, Omeprazole, felodipine, naproxen	29
P19	Male	Caucasian	67	GORD, arthritis, hypertension	Lansoprazole, Ibuprofen, amlodipine	44.2
P20	female	Caucasian	38	Polycystic ovary syndrome (PCOS), hypertension, depression	Metformin	44
P21	female	Asian	36	PCOS, hypertension, IBS	Metformin, ramipril	37.6
**Summary**	**13 Female, 8 Male**	**11 Caucasian, 4 Asian, 6 Black**	**33–71**	**Patients with co-morbidities: 20**	**Patients using medications / devices: 15**	**27.7–48.4**

### Qualitative content analysis

The primary aim of this study was to provide a qualitative exploration of the experience of weight management prior to entering a primary care led weight management programme utilising inductive qualitative content analysis. Inductive qualitative content analysis enables the quantification of phenomena and consists of the following three steps:

1. Preparation: Data collection, data review and unit of analysis selection.
2. Organisation: Open coding, categorization and abstraction.
3. Reporting: Discussion of results based on category content to describe discovered phenomena [23].

### Theme development

The first and second author read the gathered interview data repeatedly to enable immersion and gain a comprehensive, holistic picture. Next, the data were coded simultaneously by both authors to enable the creation of clear concepts and themes. Both researchers continuously made notes to capture their impressions and emerging thoughts and comprehensively discussed and examined discrepancies. From this, the primary codes emerged, which were categorized into corresponding themes. Subsequently, the final themes and sub-themes were determined to enable the analysis and discussion of the findings. The outlined steps were adapted from the qualitative content analysis approach by Hsieh and Shannon [24]. Trustworthiness was established based on the criteria developed by Lincoln and Guba to determine the credibility, transferability, dependability and confirmability of the approach [25,26].

The group of researchers involved in the creation of this study was comprised of two nutritionists, one public health scientist and one psychologist with a particular interest in weight management research. All researchers contributed to the study at varying stages from planning the study to creating the interview structure and consent forms to conducting the interviews and line-by-line coding. They continuously engaged in reflective dialogue with each other throughout the creation of this study.

### Ethical considerations

Participants were informed both verbally and in written form about the aim and format of the study, anonymity and voluntary participation prior to commencement of the interview. They were provided with a detailed consent form and contact information of the research team. The primary author discussed all aspects of the study with each participant prior to conducting the interview. All questions were addressed in detail and comprehension and consent were confirmed both orally and in written form. Participants were also provided with contact details to report any experience of negative emotions or related outcomes. This study was approved by the ethics review committee of the Faculty of Science (BETHCIE), Vrije Universiteit Amsterdam.

### Setting and format

Participants were provided with detailed information about how the interviews will be conducted and how much time they will likely need to dedicate. They were also made aware that some topics could be emotionally triggering, which might therefore require a private, uninterrupted setting. Interviews were scheduled to accommodate work and family commitments and predominantly took place in the evenings. They lasted between 35 and 87 minutes. The length of the interviews was determined by the extent of information shared by each participant within the same question framework.

All participants were provided with the same set of open-ended questions, yet were able to delve into topics they wished to include as they saw fit. Some questions were also answered without being prompted so interviews were adapted accordingly throughout. Interviews were concluded when thematic saturation was reached.

The questions each participant was asked by the interviewer were:

- When did weight first become an issue for you?
- What weight management approaches have you tried so far?
- What was your experience with each approach?
- What is motivating you to try this approach?
- What are your hopes / expectations / wishes this time around?
- How would you describe your personality?
- How have the recent events concerning COVID-19 affected your life?

The section focusing on the self-description of participants’ personalities aimed to discover if participants would describe themselves as more extroverted or introverted, easy-going or anxious and self-motivated or externally motivated. The aim was to gain insights into their individual characteristics and circumstances to enhance programme development strategies. The formulation of these topics was adapted from the five factor model (FFM) and self-determination theory (SDT) [27,28].

Once the interviews were concluded, all interviews were listened to again and short summaries were created. Next, all interviews were externally transcribed and the first author commenced line-by-line coding of the interviews according to the descriptive themes that had emerged. Upon completion of this process, the second author undertook the same line-by-line coding. Once completed, the authors agreed on the descriptive themes and the final line-by-line coding was conducted. The descriptive themes agreed on by both authors were: (1) feeling out of control in context with weight gain, (2) lack of healthcare provider support, (3) no clarity on energy balance, (4) normalisation of excess weight, (5) being surprised by weight, (5) previous success, (6) internal weight-loss barriers, (7) weight-loss motivators, (8) envisioned goal, (9) perception of overweight, (10) support from environment, (11) lack of support from environment, (12) impact of COVID-19 –positive and (13) impact of COVID-19 –negative.

Following further analysis, the final themes were developed to provide a comprehensive picture of the experience of weight management of patients entering a weight management programme. The final themes that emerged were: (1) inability to manage weight gain, sub-themes: feeling out of control with weight gain and being surprised by weight gain, (2) environmental influences, sub-themes: supportive peers and unsupportive peers, (3) weight-loss barriers, sub-themes: lack of education and external support, work, family, stress, illness and lack of readiness to change: unrealistic expectations, short-term goals, (4) personal characteristics, sub-themes: diverse personalities and inconsistent perception of overweight, (5) successful weight-loss attempts, sub-themes: various dietary approaches and increased physical activity, (6) motivators, sub-themes: health, family and self-confidence, (7) expectations and goals, sub-themes: weight-loss, improved health and better self-image, (8) weight management during COVID-19, sub-themes: an opportunity for change and a barrier to change (S1 and S2 Figs).

## Results

The 21 participants who were interviewed for this study had diverse characteristics, backgrounds and experiences. All participants had attempted to lose weight and were fluent in English. The themes that emerged were heterogeneous and multifaceted.

### Inability to manage weight gain

Participants consistently described feeling unable to manage their weight without support. They described both feeling out of control when they had experienced weight gain as well as being surprised by their unexpected weight gain. This often resulted in a feeling of powerlessness and exhaustion.

Many participants described feeling out of control with their weight gain: “I don’t know what happened. I came back [from the holiday] and I was massive, literally.” (P4) and being unable to understand how to stop or reverse this trend: “The weight just keeps going on.” (P5) Participants also described a sense of helplessness: “I just can’t seem to lose it.” (P8) “Why can’t I just click my fingers and just stop?” (P1) Some described a sense of defeat due to this experience: “I reached a point where I didn’t know how to deal with it, I suppose, so I just thought: “Well, there you go.”” (P18) This also turned into participants blaming themselves and their abilities: “During the past maybe five years, that kind of self-discipline with regard to food has broken down completely.” (P10).

A further sub-theme that emerged was that participants frequently felt puzzled by their weight gain. Many described suddenly finding themselves at a higher weight: “I don’t ever remember becoming the size I was. I simply found myself that size one day.”(P1) Some mentioned realizing that they had gained weight when coming to see their HCP: “That was only because the doctor put me on the scales and it’s like: "Well, where on earth did that come from?“” (P17), while others discovered this via photos: “I saw a photograph of me in the summer, in our back garden and I cried, and I was like I can’t do this to my child he can’t have a fat mom.” (P1) Health implications were also a factor that encouraged patients to weigh themselves: “[…] I started to notice certain things, feeling a bit tired, and I just jumped on the scales at the gym one day and I was, like: “I’m 14 and a half stone.”” (P13).

### Environmental influences

The role of social environmental influences was also prevalent and participants described both positive experiences with supportive peers and detrimental experiences with unsupportive peers. Supportive peers had the ability to induce profoundly positive impacts on the experience of weight management, while unsupportive peers could hinder or derail progress.

Supportive peers included HCPs who provided an open, neutral, non-judgmental space: “It helps to talk to someone that doesn’t really know you because they’re talking to you like a clean slate. They’re taking for who you are now. They don’t know anything about your past.” (P13) Others gained confidence, strength and motivation from their network of family and colleagues: “Because I know I can do it and I know I’ve got support from my environment, from my family, from my colleagues […]. It’s really good for your morale. It boosts your morale to do it.” (P14) Some also enjoyed having peers actively support their weight management intensions: “He checked everything that was going to pass my lips. I couldn’t have done it without him.” (P18).

The challenge of having unsupportive peers was frequently mentioned and often resulted in detrimental consequences. Participants described being made to feel guilty by family members if they did not finish their served portions: “You would always be made to feel guilty if you left food.” (P1), while others had parents who were unable to provide healthier food options: “When I was growing up, mom used to feed me a lot of convenience food. My mum did a lot of shift work, she used to do nights and stuff like that. It was mainly just because of convenience, really.” (P16) Some encountered active resistance when trying to lose weight: “If ever I broached the subject of I want to go on a diet: "You don’t need to go on a diet.”” (P1) and were questioned when doing so: “They always doubted me when I told them and they all said to me: "Why are you losing weight?””(P11) The notion of growing out of their weight eventually was also repeatedly mentioned: “Obviously, as a girl going through puberty and stuff, and my family always use to say: "You can’t be fat. You can’t be fat. You’ll grow out of it," and I just never did grow out of it.” (P20).

### Weight-loss barriers

The main weight-loss barriers encountered by participants were a lack of education and external support as well as a lack of readiness to change. The lack of education mainly presented itself in a lack of knowledge and skills around food and behavior change. Participants felt that they had no clarity on how sustainable weight-loss can be achieved and HCPs did not frequently offer support or guidance. Some also found it hard to commit to a programme long-term due to discouragement or circumstantial changes.

Lack of education presented itself in various misconceptions about weight-loss from food groups: “I’ve cut out so many things in my diet, but it doesn’t seem to make a big difference.” (P8) to meal timing: “I’m probably not eating at the right times at the moment.” (P3) Participants often felt they had exhausted all resources according to their knowledge in the pursuit of weight-loss: “I don’t actually know why. I was doing everything I should of.” (P13), even resorting to potentially harmful options: “I literally was just sitting there and I said to him: "Have you got a pill that I can take because I’m not losing weight? I’m doing everything, but I’m not losing weight." (P4) Patients were frequently met by unsupportive HCPs: “The doctor, who was horrendous, he always just said: "It was because you’re fat. […] Any time I went to the doctor, I would think: "If you weren’t so fat, it would help." He never said: "Oh, let’s do this, let’s do that.”” (P20) even when diagnosed with weight related co-morbidities: “He just basically came out, and he was like: "Oh, you’re diabetic," and that was it. […] “You better start taking pills." I was like: "No." I resisted for a month.” (P4).

A lack of readiness to change often expressed itself in participants finding it hard to stick to weight-loss programmes and approaches. This was frequently underpinned by unrealistic expectations and a short-term focus: “The problem was it was always on and off. It was never a frequent pattern. […] I may have dropped a little bit, but then it probably went up again because I thought to myself: "Oh wow. Look, I’ve dropped a little bit of weight. Hey, what difference is it going to make to my life really?”” (P4) Participants also found it hard to implement change without external support: “I’m aware but I cannot do anything because I think I need more encouragement. […] I’ve got so many excuses.” (P14) and some prioritised taking care of others instead of themselves: “I was busy looking after other people at that time, so I didn’t think about myself too much. […] Rather than I suppose really try and do something about it, I felt I didn’t have time. I was too busy with the children, and then, I was looking after grandchildren, and then it was mum, and it was just—I really put myself on the back burner. I put everything and everybody else first.” (P18).

#### Barriers resulting in weight regain

Busy work schedules and professional commitments frequently resulted in weight regain: “You’re working longer hours because you have to produce the results and all this, etcetera. You forget about exercising and all of a sudden you start putting on the weight gradually, gradually, gradually.” (P15) driven by sedentary behaviours and social obligations: “[…] I was working 14, 15 hour days in an office sat down. You’d be out drinking every single day with clients or with colleagues.” (P1) Participants also regained lost weight due to illness: “Then I had problems with my stomach which meant I had to stop going to the gym.[…] I sometimes wasn’t able to eat for periods of time and then when I was able to eat, I just ate anything I could because I was so hungry.” (P7) Social occasions, new relationships and a lack of prioritisation also frequently lead to weight regain: “There’s Christmas coming up, or there’s Easter. It’s someone’s birthday […]. Once you make that bad hiccup, you fall off the wagon […].” (P13) “We probably had two years of eating, drinking, and just enjoying the first couple years of our relationship.” (P6).

### Personal characteristics

The personal characteristics of participants were very varied and diverse. This strongly highlights that participants have individual, multifaceted needs and should therefore be understood within their reality. An inconsistent perception of overweight was also observed with varying degrees of intrinsic acceptance of overweight.

Some participants described themselves as shy and reserved among new people, yet open when comfortable: “I get a bit nervous now meeting new people. […] If you get a good vibe from people, I’m just me.” (P13), while others described themselves as extroverted:” If I have time, I would socialize every day. I love talking to friends, to my colleagues. It’s good for your well-being.” (P14) The majority described themselves as being both extroverted and introverted, depending on the situation: “I like both, if I’m honest with you. I have moods where sometimes I want to be with everyone, but then sometimes I want to be on my own, I want to do stuff by myself.” (P4) Some participants described themselves as quite anxious at times:”My anxiety is overthinking things, worrying about things that haven’t even happened yet.” (P13), while others felt they were more easy-going:”I’m easy-going. I go with the flow. I can lead, but I’m quite happy just going where the will and the wind takes me.” (P17) Most participants described themselves as being both anxious and easy-going depending on the situation: “I suppose I’m pretty easy-going, but I do get anxious about silly things […].” (P18) Some participants described finding it easy to motivate themselves: “Well, generally, I’m more self-motivated in respects to the weight-loss side of it as well.” (P3), while others described relying more on external motivation: “I think where it’s something about I am not left outside of my comfort zone, then I definitely will rely on other people.” (P6) The majority of participants described being both self-motivated and relying on external motivation, depending on the situation: “If I need a push I would need someone else to push me, but I can also pick myself up to get on with stuff if I need to.” (P2).

Participants also described different perceptions of their own weight. Some felt ashamed and insecure due to their weight and described experiencing excess weight as a burden: “It looked ugly, persons who don’t look ugly immediately when they put on weight, but I was one of those who just got a round waist and I started looking ugly and I didn’t like that.” (P10) “You feel insecure when you go out with your friends because they can wear sleeveless, they can wear backless. […] Then you cover yourself, so now you’re more aware of your body image.” (P14) Conversely, others were not as preoccupied with their weight: “It never hindered me. Obviously when you’re young, you don’t really think about it, to be honest.” (P4) Some also accepted their weight: “[…] I was always quite big anyway, I was never a skinny person. […] I suppose I couldn’t do as much as I used to, but it didn’t worry me. I didn’t feel ill. I felt okay. I didn’t have problems. It didn’t worry me at all.” (P19) and felt this was their only option: “I couldn’t lose it and I suppose from then I just accepted, "Oh well, that’s it. I’m this weight now," and that was it.” (P18).

### Successful weight-loss attempts

The majority of participants described having had various temporarily successful weight-loss attempts in the past. These were achieved with the help of a wide range of dietary and behavioral approaches often in combination with increased physical activity levels.

Approaches were very varied and included mindful eating: “I was watching what I was eating and maybe I was walking more. That’s when I managed to lose some weight.” (P12) combined with healthier food options: “I probably ate less, but I also ate more of the right things” (P19) or calorie counting: “At that time, we started using Noom [app] and we got used to it very, very well. […] I just counted my calories, that’s it. “(P21) Others resorted to unhealthy approaches: “[…] I just survived off of black coffee, Lucozade and Marlboro Light cigarettes.” (P7) and weight loss medications: “Then I tried slimming pills […]. That was really easy. I was like: "Wow. You just never want to eat.”” (P5) “Well, I then started diet pills to get myself a bit of confidence and I got myself back down to about nine stone.[…] They were from a place where I used to gamble. So probably not certified.” (P1) Some also developed less healthy eating habits to achieve weight-loss: “[…] it became very easy for me to stop eating altogether, and that’s almost what I did […]. (P1) Several also joined slimming clubs: “Before that, I had tried to do Slimming World on two occasions […]. I enjoyed it.” (P13).

In order to increase physical activity levels, several participants took up walking: “I did so much walking like I haven’t done for the last five years […].” (P15) “[…] I walked a lot. […] Maybe every day I walked more than an hour.” (P21) and started going to the gym regularly: “I did join a gym, and then that was going quite well. I did lose some weight, and I put some of the muscle back on. […] I went on average about three times a week.” (P17) “[…] I started doing exercise classes […]. I would do circuit training, I would do step aerobics, I would do spinning, you name it, I would do it.” (P1) and got support from personal trainers: “I started going to the gym when I was probably around 25 and that was really quite good actually. […] I got a personal trainer, so whatever he told me to. […] I would go to the gym five times a week.” (P1) “A friend of mine was a personal trainer. I went to see him twice a week after work.” (P6) Some also joined groups: “We did join a diet group and we would exercise once a week. I did lose quite a bit on that.”(P16).

### Motivators

The primary motivators were consistently mentioned to be the achievement of improved overall health, the desire to be able to have various experiences with family members and increased self-confidence.

Participants frequently mentioned having family members with chronic diseases: “I decided for myself and you have a history of diabetes in the family, high blood pressure. I think I should really take care of myself now.” (P14) and emerging, weight related co-morbidities: “Sometimes I have this reflux of the fatty foods that you eat.” (P14) “Then he said that I might have COPD, but then he said it might be, I have too much weight pushing my lungs up or stuff like that. […] The other thing is my walking because I had that breathing problem. (P15), which impacted quality of life: “[…] first thing in the morning my knees are quite sore which again, has been quite a wake-up call and it’s something that you don’t want in your 30s.” (P2).

Participants were also mindful of wanting to be present in their family’s lives: “Obviously I’ve got kids. I’ve got young kids. I don’t want to have a problem later on in life. Obviously, when I had kids, I […] started thinking differently. It’s when I was coming up to 40, I was like, "No, I need to make a difference because if I don’t, then obviously my kids will suffer.” (P14) “Family certainly are without a shadow of a doubt […].” (P3) Participants expressed wanting to be alive and agile long-term: “I want to live for a good long time. I want to be able to move and see my grandkids […].” (P1) and hoping to conceive: “We want to start a family it’s obviously impeding on that." (P2).

In terms of self-confidence, motivators were both extrinsic and intrinsic in nature: “I wanted to look better because I love wearing dresses, I love going out. I’m presenting in front of so many people now. I have to speak in public, so I have to be presentable. If you’re a good example to your staff and to patients, they will believe you. We have so many patients with diabetes and then how can you teach them if you’re the one who is not?” (P14) Some were motivated by images: “It was seeing pictures of myself and then realizing that I wasn’t very happy with myself and my body image.” (P2) and others had reached a turning point: “I thought: "I need to do this for myself and my own self-esteem and for my family.”” (P17) for some due to social implications: “From December, January, I stopped going to parties over here also because I knew everyone is going to comment on my weight, number one. Number two, none of my clothes fit, the party clothes, and I didn’t want to buy anything new. That was another reason.” (P21).

### Expectations and goals

The primary expectations and goals were comprised of weight-loss, improved health and better self-image. Participants felt that all three were intrinsically linked and would inevitably lead to each other.

Participants mentioned the desire to be in the healthy weight range: “My goal is to be within the healthy BMI range.” (P12), often combined with specific weight goals: “I think I’d like to get my BMI between 26 to 28 max, and probably my weight, I’d say 12st 7 I think, is reasonable at the moment.” (P2) “If I get to 10 stone which is what I weighed when I was nine months pregnant I’ll be more than happy.” (P5) In terms of health improvements, participants mentioned overall health improvements: “What I want is just to be healthy.” (P14), improving specific conditions: “I want the doctor to say to me: "Look, you’re not at high risk anymore for getting diabetes.”” (P13) “I know if I nail one, I nail all three [T2D, hypertension, hypercholesterolemia] […]. That would be probably my main aim, my most crucial aim to make sure I could maintain that and control it.” (P3) “Number one, was my diabetes.” (P18) and overall agility: “I want to be able to move and see my grandkids […]”. (P17) “Get a little bit fit. Walk a bit more fast […] If I can walk a little bit faster and breathe a bit more.” (P15) Participants were motivated by the impact weight-loss would have on their self-image: “[…] to feel better about myself. […] I can wear nice things, and I feel attractive in it.” (P18) “It’s more about how I look and feel.” (P6) “To be more self-confident.” (P1).

### Weight management during COVID-19

COVID-19 had a profound impact on participants in terms of weight management, with some experiencing it as an opportunity for change, while others experienced it as a barrier to change. This was highly dependent on personal circumstances during COVID-19, ranging from health to housing, work and childcare.

Some participants felt that COVID-19 gave them a chance to focus on their weight-loss goals without external distractions: “I’ve been going for walks more, I have- obviously, not going out with friends for dinner. That’s been really helpful because of the fact that I thought it’d be so easy to slip into drinking every day like everybody else has”. (P1) “Yes, it’s been a positive experience in two senses. A, it has facilitated that it’s easy to stick to your prescribed diet, because we’re not being invited to dinners. […] B, I’m doing more sports. For the first time in a long period if at all there was an earlier period like that, I have a well-regulated circadian rhythm which I haven’t had before.” (P10) Participants mentioned having more time for cooking: “I mean, food-wise we’ve been quite lucky. We’ve been able to get our shopping delivery […]. We’ve been looking through cookbooks and bits and pieces and being inventive with whatever turns up.” (P17) and finding that it helped them stick to a programme better: “I’ve sort of used it to my advantage, the COVID, it’s made it a lot easier. It’s also made it easier because I haven’t had people commenting on what I’m doing.” (P5).

Others experienced COVID-19 as a barrier to change due to heightened anxiety: “It’s been awful. I’m not going to lie. I’ve had to ring my doctor and ask for tablets just to take the edge off things […]. Look, you’re having a bad time as it is, just eat what you want." (P13), frequently combined with loneliness: “I felt very anxious, felt very worried. I’ve also felt very helpless […]. I felt quite lonely sometimes as well, where my husband has been out for such a long period of time and just having an eight-year-old say "Mummy, mummy, mummy, mummy, mummy, mummy, mummy, mummy, mummy” […]. I’ve just wanted to eat constantly.” (P1) Some had frontline jobs with increasing workloads due to COVID-19: “It’s been a bit of a rollercoaster since COVID has hit. I think working very long days, and with buying food at work […], coming home completely exhausted and just needing something to pick me up a little bit […]. There was an element of emotional eating because it was really worrying.” (P2) Changing childcare arrangements and cooking for the whole family were also perceived as barriers: “I think it’s obviously the boredom and the fact that I’m at home cooking the kids or preparing 25,000 meals a day. The fact that I’m just at home is not a motivation to not eat […]. Obviously, being a single parent, I feel quite isolated, because I have no one else here. I don’t have anyone.” (P20).

## Discussion

### Main findings

This study set out to create a qualitative exploration of patient experiences with weight management prior to entering a weight management intervention programme. The goal was to gain insights into the experience of weight management and the resulting consequences encountered. Research into the experience of substantial, sustainable weight-loss maintenance has continuously found that individuals with overweight and obesity experienced the achievement of substantial, sustainable weight-loss maintenance to be challenging due to varying factors including everyday stress [29–35], emotional eating and boredom [33,34,36,37], planned life events [14,29,30,38,39] as well as unplanned life events [29,33,34], amongst others. Environmental influences have also frequently been cited as potentially derailing factors including discouraging peers [30–32,39–41], the obesogenic food environment [14,30] and the inability to stay motivated without support [36,42]. A lack of HCP understanding and support has also consistently been experienced as a barrier [18,43].

Motivators generally include improved health [14,30,33–35,37,39] and self-image [14,29,31,34,35,41,44,45]. Studies have also highlighted the desire to decrease the experience of weight stigma as a motivator for weight-loss [34,37]. Research into substantial, sustainable weight loss maintenance has highlighted that weight management support is instrumental for helping patients achieve substantial, sustainable weight-loss maintenance [46–49]. The present study and the systematic review by the authors [21] confirm these findings. Notably, the motivators appear to be similar across participants yet the variances in personal characteristics highlight the importance of individually tailored care to help achieve treatment success. Continuous research with a focus on patients’ perspectives and experiences can enhance treatment quality. This perspective also has the potential to decrease weight stigma among HCPs working in the field of weight management.

### Strengths and limitations

The strengths of this study include gaining in-depth insights into the experience of weight management in a cohort that is diverse in terms of ethnicity, age, gender, health status and BMI, which facilitated data saturation. The structure of the interviews allowed extensive exploration of a broad variety of encountered experiences. Limitations include the inability to conduct the interviews in person due to COVID-19. Additionally, the personal characteristics were self-defined by the participants, which may have been influenced by their subjective self-perceptions. Memory recall bias is a further noteworthy potentially limiting factor as participants may have altered recollections of experiences [50]. Therefore, this study has to be seen in light of the mentioned limitations.

### Implications for research and practice

This study provides important insights for both weight management research and practice. Individuals with overweight and obesity often feel unable to control their weight on their own due to a multitude of factors and can greatly benefit from external education and support. Patients with overweight and obesity tend to be highly motivated to lose weight as well as gain better health, improve their self-image and feel that sustained weight-loss will help them achieve this. This study highlights that, while personal characteristics and experiences differ, motivations and goals of individuals trying to achieve weight-loss are mostly aligned. Notably, motivations were predominantly extrinsic in nature. According to self-determination theory, beneficial long-term health outcomes are usually linked to intrinsically driven motivation, highlighting a potential barrier for long-term success [28]. Research can build on the findings of this study and others of its kind to provide further insights and enhance treatment quality and outcomes. In practice, HCPs can play a significant role by creating personalised treatment protocols for individuals aiming to achieve substantial, sustainable weight-loss maintenance.

Participants frequently mentioned a lack of HCP support as well as experiences of weight stigma, which is an area worth highlighting. Weight stigma can have detrimental implications on the physical and mental health of individuals as well as deter patients from seeking medical care [51] and is therefore an important area to address. Obesity is currently classified as a chronic disease in 5 countries and by the WHO [52–55] and patients with overweight and obesity can benefit greatly from comprehensive treatment and HCP support. Studies have shown that, while patients with overweight and obesity infrequently get comprehensive weight-loss support from HCPs, it usually increases both motivation and desire to do so, irrespective of degree of overweight, age and gender [18–20]. The present study and others of its kind highlight that individually tailored solutions focusing on modifying both attitudes and personal environments have the potential to enhance treatment outcomes. Obesity is a multifaceted condition that will require comprehensive engagement from HCPs to be managed effectively. Trends are continuing to increase globally and continuous research and clinical support will be required to help reverse this trend.

## Conclusion

This study highlights the importance of gaining a comprehensive understanding of experiences individuals with overweight and obesity encounter when trying to achieve weight loss as well as understanding their individual expectations upon commencement of an intervention. It became evident that the need for comprehensive support is strong as patients describe feeling unable to control their weight without external support, yet were highly motivated to lose weight to improve their health and self-image. Personal characteristics and experiences were diverse but the desire to lose weight was consistently prevalent. Gaining a deeper understanding of the diversity in support required by patients due to their individual, unique experiences and circumstances through meaningful conversations has the potential to significantly improve long-term outcomes. Participants also encountered a lack of support and weight stigma, which highlights the importance of further training and education for HCPs to improve treatment strategies and reduce the prevalence and impact of weight stigma.

## Supporting information

S1 Fig Lifecycle of weight management. This figure encapsulates the themes identified when investigating patient experiences when trying to achieve weight-loss prior to entering the primary care-led weight management intervention.
(TIF)

S2 Fig Theme prevalence cohort. This table illustrates how frequently the theoretical themes were organically discussed by all participants broken down into percentages out of a total of 100% to determine the prevalence of each theme during the interviews. Due to variability in responses, recollections and communication style, the prevalence shown in this graph serves solely illustrative purposes.
(TIF)

S1 Appendix Supporting information–appendix.docx. Interview quotes from qualitative dataset.
(DOCX)
