# Supplementary figures and images for: Patient insights into the experience of trying to achieve weight-loss and future expectations upon commencement of a primary care-led weight management intervention: A qualitative, baseline exploration
Source: PLoS One. 2022 Jun 29;17(6):e0270426. doi: 10.1371/journal.pone.0270426 (PMC9242434; doi:10.1371/journal.pone.0270426)

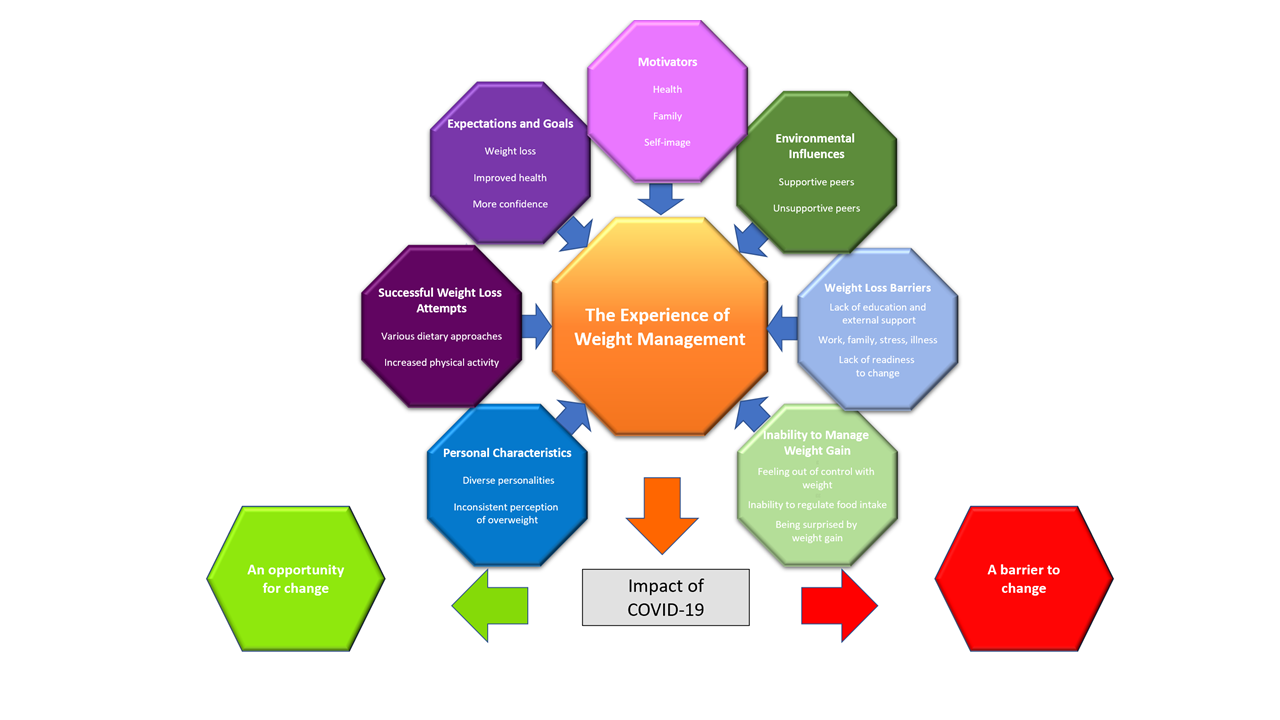

Supplement: S1 Fig — This figure encapsulates the themes identified when investigating patient experiences when trying to achieve weight-loss prior to entering the primary care-led weight management intervention. (TIF) [file pone.0270426.s001.tif]

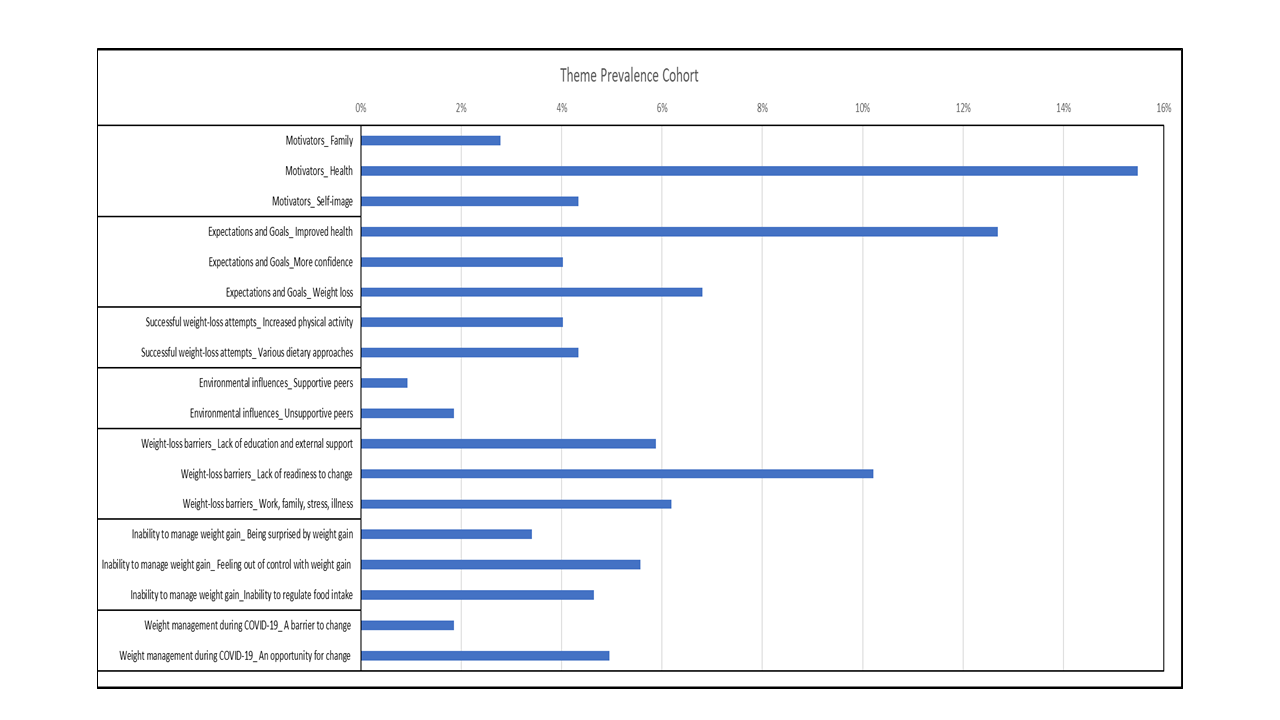

Supplement: S2 Fig — This table illustrates how frequently the theoretical themes were organically discussed by all participants broken down into percentages out of a total of 100% to determine the prevalence of each theme during the interviews. Due to variability in responses, recollections and communication style, the prevalence shown in this graph serves solely illustrative purposes. (TIF) [file pone.0270426.s002.tif]
