# Supplementary material for: Patient insights into the experience of trying to achieve weight-loss and future expectations upon commencement of a primary care-led weight management intervention: A qualitative, baseline exploration
Source: PLoS One. 2022 Jun 29;17(6):e0270426. doi: 10.1371/journal.pone.0270426 (PMC9242434; doi:10.1371/journal.pone.0270426)
Supplement: S1 Appendix — Interview quotes from qualitative dataset. (DOCX) [file pone.0270426.s003.docx]

# Appendix

| **Theoretical Themes** | **Subthemes** | **Supporting Data** |
| --- | --- | --- |
| **Inability to manage weight gain** | Feeling out of control with weight gain  Inability to control food intake | “I don't know what happened. I came back and I was massive, literally” (P4) “[…] sometimes when you do the job and you have to produce results and all that, et cetera, because that took over me I think that's what happened really, to be honest “ (P15) “It's just one of those things. Like, you know that you shouldn't take drugs, but you kind of still do.“ (P6) “I would eat everything and anything I could get my hands on.“ (P1) “I just put my weight on and weight on and weight on” (P19) “I just can't seem to lose it.” (P8) “I reached a point where I didn't know how to deal with it, I suppose, so I just thought, "Well, there you go." / . I couldn't lose it and I suppose from then I just accepted, "Oh well, that's it. I'm this weight now," and that was it. I just carried on.” (P18) “[…]the weight just crept on, crept on, crept on.”” it just kept going up, and up, and up” (P13) “just seemed to just start from there when it really started to pile on really” (P16) “[…]the weight just piled on” (P11) “[…]and bang, I put all my weight back on.” (P20) “I just put on weight.” (P2) “Then the weight slowly crept back up, back up, back up. / I feel like I let myself down all the time. / With weight. I know what I need to do. Why can't I just click my fingers and just stop? Why? What gives you will power some days and then not others? / [unintelligible 00:48:25] put it on. Why is it so easy to put it on?” (P1) “The weight just keeps going on.” (P5) “During the past maybe five years, that kind of self-discipline with regard to food has broken down completely” (P10) “It was going up and up and up because I was just continuing eating. I thought that I didn't have any other option. The only way I could cope with the stress of work and the frequency and everything was eating” (P21) “I got just into this curve of up, and up, and up and I couldn't stop it, which is really not nice” (P9) “There were times when I would literally go up and down and it's hard to believe. ““In the course of the 12 months in a year, my weight could fluctuate immensely. I could be at one stage two stone heavier than I was.” (P3) |
|  | Being surprised by weight gain | “I don't ever remember becoming the size I was. I simply found myself that size one day.“ (P1) “I remember working it out. It worked out like 19.99 stone. Something like that, 19. I was like, "Fair enough, that's incredible." (P6) “I don't know. I must have put on weight. I didn't even realize I've put on weight that much in two months or whatever.” “I don't know what happened. I came back and I was massive, literally” (P4) “I remember we all introducing ourselves and speaking to each other as a group and you were happy to talk about your ages and things like that, and I thought, Blimey, what had I done to myself to get here because genetically I was always going to be predisposed to higher blood pressure, diabetes, things like that.” (P3) [Interviewer: Was it surprising to you then when you started gaining weight?] ”Yes, and then I have to change the sizes of my dress, my jeans, everything. I cannot use my jeans anymore. It's size 14 to 16 and then it's so depressing when you reach 18. I said to myself, I don't even want to look at the mirror because body image is different. (P14) “Yes. I don't know why.” (P19) “I didn't kind of notice it as such, not until Christmas last year when we came to see you. That was only because the doctor put me on the scales and it's like, "Well, where on earth did that come from?"” (P17) “In my 50s I think it seemed to all of a sudden, "Oh bang. Where did that come from?" Actually, I think I just ignored it, which wasn't a good idea. Then, I've seen some photos going back a little bit and I'm looking at myself, "Oh, I'm definitely overweight."” (P18) “Yes, I started to notice certain things, feeling a bit tired, and I just jumped on the scales at the gym one day and I was, like, "I'm 14 and a half stone."” (P13) “I really don't know why I'm so fat, because I was just saying this to my friend today, the job that I do is so physical and all the manual handling and up and down on the floor and the change in blah, blah, blah, pushing wheelchairs. Obviously, at home, I don't stop either. Every time I go to sit down, I'm standing back up.” (P20) “I saw a photograph of me in the summer, in our back garden and I cried, and I was like I can't do this to my child he can't have a fat mom.” (P1) “[…] none of my clothes were fitting. I didn't have any clothes to wear in that gathering. I had to buy clothes before going there, and I noticed that I had to buy a size 18-20. That was quite shocking for me. (P21) “I did not notice that I did put on so much weight. Only now as I'm reducing my weight with your program, I now remember the feelings what I was feeling like when I was slimmer / Now looking back, I am surprised to see that I put on weight and I lost my confidence obviously the last 12, 15 years. (P9) |
| **Environmental Influences** | Supportive Peers | “It helps to talk to someone that doesn't really know you because they're talking to you like a clean slate. They're taking for who you are now. They don't know anything about your past.” (P13) [Interviewer: Did your husband motivate you?] “Yes, it was. My husband had lost quite a lot of weight from walking to work and he picked up on the fact that I hadn't so we had those honest conversations.” (P2) “I remember my aunt suggested to me, "Play netball and do like a run," so just-- I can't remember how old I was, about 25, something like that. My dad got me a Wii Fit, like the Wii Fit boards. He got one for himself. My aunt, unintelligible 00:06:36] so she was doing it, and she was like, "I've lost a little bit of weight doing the Wii Fit, and stuff" “The conversations would just be around eating, drinking, over indulging, but there were conversations in place for advice, and we know we said, "Right, you need to stop eating this because this is what's making you fat" or "You need to stop drinking"” (P6) “The first thing was my cousin. She always told me that if I lose weight, then all my problems are going to go away. When she started with you and she was so happy, she was so happy and so convinced and she felt like it was the life-changing thing for her. When she introduced me to you after the first time we talked, she called me and then she said that I should be happy because all my problems are solved now. I got introduced to you and I got enrolled into this program so I will lose weight and then I won't have any problems in my life anymore. That's how she convinced me.” (P21) “We've all joined the gym even though now, we're on lockdown. We've still got our membership and we're going to get back to that when things are back to normal, which I'm looking forward to. I feel that this has brought us closer together as a family, we're doing more activities together.” (P16) “Because I know I can do it and I know I've got support from my environment, from my family, from my colleagues […]. It's really good for your morale. It boosts your morale to do it.” (P14) “[…] he was a great motivator and encouragement during that time” “He was very impressed, and it was nice because he was spending a lot of money to have me on this diet, and he kindly did that. It was nice for him to see the rewards of it all.” (P11) “I only had to go to me mom and say, "I've got to lose weight and all this sort of thing." She'd go, "Okay, then that's it. You can't have this, you can't have that and this and that," and she knew all about how to do it because she had to do that for diabetics in a diabetics' home.” (P19) “He actually joined me. He didn't go as low as I was obviously. He's still at work and he needed to look after himself to then do his job. He cut down and we had brown bread all the time. He checked everything that was going to pass my lips. I couldn't have done it without him.” (P18) |
|  | Unsupportive Peers | “They would find a pub near to my gym to go for a drink. As soon as I came out the gym, my friend would be ringing, start buzzing. I would basically leave the gym and then have a couple of pints with them.” (P6) “Which is quite funny because friends thought I would fail. When I saw her- she looks, she goes, “I thought you’d fail. I didn't think you'll stick to it.”” (P4) “When I was growing up, mom used to feed me a lot of convenience food. My mum did a lot of shift work, she used to do nights and stuff like that. It was mainly just because of convenience, really.” (P16) “People can be very judgmental. If I was going into work, "Oh, what are you're having for lunch?" Then you have to explain. "Oh, you shouldn't do that." That's all you ever get whatever diet you're on. I always find that people have got their opinion. They either want you to do their diet or they think whatever yours is doing is completely wrong. That never helps” (P3) “You would always be made to feel guilty if you left food.” (P1) “I had conversation with my parents about that, but I don't think it went anywhere beyond a conversation really.” (P12) |
|  |  | “I think it was always one of those, "Oh, you know what, as he gets older, he'll probably lose it, baby fat kind of thing." It wasn't like, "Oh, you need to worry about it," anything like that” (P4) “I think kind of a throwback for my mum and being Italian, everything was pile on the plate, "You are a growing lad, you have to eat."” (P17) “I'd put it down to just sheer contentment. I was getting married to someone I thought looked tough and it was just perfect, and they said that's what that is. That was my grandma's view.” “A lot of people said to me, "Oh, it's the middle-age weight and stuff."” (P18) “I think everyone just accepted that I'm just the way I was.” (P16) “Obviously, as a girl going through puberty and stuff, and my family always use to say, "You can't be fat. You can't be fat. You'll grow out of it," and I just never did grow out of it.” […]”and I was being told, "It's puppy phase. It's puppy fat. You'll grow out of it. You'll grow out of it" (P20) “almost being in that generation where it was okay to do that and not worry too much about the gym or running or what have you.” (P2) “If ever I broached the subject if I want to go on a diet, "You don't need to go on a diet."” (P1) “It's a game that I think it's part of finishing the past year. The bride and groom, they all just gain weight just because of all these invites and everything.” (P21) “Obviously, a very nice colleague of mine said so many people, it happens that they put on weight.” (P9) “[…] people would say, "No, you're not big," and, "You're fine." I don't know whether I have always been surrounded by nice people or not. (P12) “They always doubted me when I told them and they all said to me, "Why are you losing weight?””( P11) |
| **Weight-loss Barriers** | Lack of education and  external support  Work, family, stress, illness | “I don't know what it was because I didn't eat any meat or anything. My family are vegetarian so there was no eating fast food and stuff like that when I was out there.” “Just eat, go to the gym. I thought if I'm going to the gym, going swimming, I'll start to lose weight. It's not really something I thought about. I ate less, yes, but I wouldn't say I was counting calories because, Marie, I'm 25. I don't care.” “I literally was just sitting there and I said to him, "Have you got a pill that I can take because I'm not losing weight? I'm doing everything, but I'm not losing weight." From last year, I was hitting the gym constantly. We're talking three, four times a week. I was going to the gym, but the weight just was not moving.” (P4) “The thing with the nutrition, and it's hard to believe sometimes, when I do eat, I actually eat very healthily [laughs], when I do eat.” I'm probably not eating at the right times at the moment.” (P3) “You start getting in the habit where you start having a big breakfast and in ruins my-- What do you call that then? Your eating plan per day, where you have a big breakfast and you work during the day and you don't even eat nothing.” (P15) “I've cut out so many things in my diet, but it doesn't seem to make a big difference.” (P8) “I don't actually know why. I was doing everything I should of.” (P13) “I obviously had a very high metabolism until I interfered with it by drinking these build up drinks as they were. It ruined my metabolism but prior to that, I did not have a problem with what I ate, what I put into my mouth.” (P11) “I really don't know why I'm so fat, because I was just saying this to my friend today, the job that I do is so physical and all the manual handling and up and down on the floor and the change in blah, blah, blah, pushing wheelchairs. Obviously, at home, I don't stop either. Every time I go to sit down, I'm standing back up” (P20) “All I did was skipping meals and eating mostly egg white, because I had read that egg whites is burning fat / I cut out sugar almost completely. I stopped eating fruit and drinking fruit juices, because they all have fructose in them and fructose is a dangerous sugar.” (P10) “Then I had problems with my stomach which meant I had to stop going to the gym. I think that once I stopped going to the gym, I started to become ill and I sometimes wasn't able to eat for periods of time and then when I was able to eat, I just ate anything I could because I was so hungry. I just completely lost that balance which I found and then the weight just started piling back on again into my mid-30s, really.” “I was going to carry on going and then, basically, just got really ill. That, basically, it cocked everything up and took me back to almost where I'd started. Not quite where I started [crosstalk].” (P7) |
|  |  | “I don't think I did any programs or anything. No one offered me. No one said anything to me.” “It wasn't like, "Oh, you're diabetic." I think it was my Doctor who was there before my current one. He just basically came out, and he was like, "Oh, you're diabetic," and that was it. There was no, "Oh, you need to lose-- Let's see if you can lose weight or do something to try and counter it." It was literally, "You're diabetic. You better start taking pills." I was like, "No." I resisted for a month.” (P4) “No.[…] Oh, how can I say it? It was more like just telling me, "But it's up to you." It wasn't like, the support you're giving me. It was just like face value and then you do the rest and all that. There was no backup. It was like, "Go to the gym." Some leaflets. What you're eating. "This is what you've got to eat." That's it. There was no logistical support that you needed.”(P15) “No, not from healthcare providers.” (P6) “Nobody said about losing weight. My Dr. said once about if I was to lose weight with my arthritis, I would feel better but nothing was set in stone or anything like that” (P19) “Once, yes. Have a look, "Yes, your diet is okay. Cut down the sugar and the milk." That was all they said.” “It was completely unhelpful and utter waste of time.” (P17) “I said to him, "How long has this been going on?" "Oh, a few years. It's only crept up." I said to him, "Why didn't you tell me before we got to this stage. When it was noticeable, why didn't you tell me in the beginning?" He says, "What would you have done about it?" I was astonished, to be honest.” (P18) “No, I've not-- No one really ever. / No, I was never offered any help.” (P17) “No. I went to a dietitian once. I did go to a dietitian once through a doctor. The dietitian, he was quite negative.” “It was all about you, you, you, you. I didn't find it supportive. You'd been trying, and you'd been taking these changes, but then that never seemed to be good enough. It was, "You got to have [unintelligible 00:15:25]. You got to have new potatoes now." “I just felt that it was never good enough” “I went to a few appointments, and, again, he just was like, "Well, if you want this, because it's classed as cosmetic, you've got to lose X amount of weight." He was quite arrogant consultant, really. I didn't find him very nice.” “I used to go to the GP to go and see the doctor about something-- [unintelligible 00:17:23] and [unintelligible 00:17:23], whatever. The doctor, who was horrendous, he always just said, "It was because you're fat." [Interviewer: He just said that to you?] “Honestly, yes. He was like, "You're just fat." Any time I went to the doctor, I would think, "If you weren't so fat, it would help." He never said, "Oh, let's do this, let's do that."” (P20) “I genuinely don't think healthcare professionals have time to devote to actually doing what's needed and yes, I've had my GP tell me that I need to lose weight and that's more because I went to them about fertility and the fact that things weren't happening. I just don't think they have the time to actually sit and delve into things unless they need to, or should be able to” “[…] you're sent away to just deal with it yourself and that is quite frustrating, but it is limited resource, so I do understand. “(P2) “No. No. This was the first time” “Nobody's ever said to me any way of being-- Whether it's been a nurse when I've been for a smear, or doctors, "Would you like any support? We've got these programs?" No” (P5) |
|  | Lack of readiness  to change:  Unrealistic expectations  Short-term goals | “The problem was it was always on and off. It was never a frequent pattern.” “I may have dropped a little bit, but then it probably went up again because I thought to myself, "Oh wow. Look, I've dropped a little bit of weight. Hey, what difference is it going to make to my life really?" Do you know what I mean? I'm diabetic already. I've just got to be careful what I eat.” (P4) “No. No, I did but I didn't actually do it. I really want to do the keto diet but I cannot because I'm working. I really need to sustain myself so I have to eat. Because I cannot function without food, especially carbs” “'I’m aware but I cannot do anything because I think I need more encouragement. I cannot go to the gym. I was so busy at work. I'm working Monday to Saturday and I only have one day off during the week. I've got so many excuses.” “It's trying to gratify yourself or making yourself happy by eating.” “You just eat what you wanted to eat. You deserve it, because I worked hard for it.“ (P14) “Well, but what I do do is that I eat very, very late at night, so I don't do anything after that. Then I drink a lot, and I had been drinking a lot throughout those years. I wouldn't say I was an alcoholic, that's a bit harsh, but when I binge drink, so on a Friday, Saturday on the weekends, over that Friday, Saturday, Sunday, I could easily consume 20 pints or 20 cans, I should say.” ”If there's a pack of 24 in a pack, I could easily consume that, and particularly with family, friends, we’re very social as a family. If there's lots of sports events, we'll all get together to watch or whatever it might be. Those weekdays, you're at work, you've got all those commitments during the week, you don't do anything, but come Friday night, Saturday night, Sunday afternoon, it's then [unintelligible 00:11:03], and that's been a huge habit to have to try to break.” “Consequently, without a shadow of a doubt, I am very weak in that respect, so it's been a perfect storm from not eating throughout the day, it's only in the evenings, then the beer on the weekends and the binge drinking on the weekends.” (P3) “I was able to keep it controlled, and then, I know it's the easy way out when you get married. Married life, and looking after.” “Obviously, when you get into cooking and all that, et cetera, obviously you're going to eat more and all that. Also, at the time, we'd go to family parties or get-togethers and all this, et cetera. You're putting on the weight during the weekend and all this, et cetera. I wasn't exercising as much.” “You're working longer hours because you have to produce the results and all this, et cetera. You forget about exercising and all of a sudden you start putting on the weight gradually, gradually, gradually. You don't think that you're eating too much on the day but you are” (P15) “Three years of just really a lot of drinking. A lot of drinking and not a lot of exercise. Not probably a very good diet as well, fast food and oven food, basically just eating whatever you could. I started to put on weight throughout uni. / I've joined so many gyms over the years and I will just pay every month and not go. I must have joined five or six gyms over the last 10 years--/ After that, different jobs I'd had or different roles, I signed up to gyms that were near a job. Again, I really struggled with that motivation to go, to just get me in there. That never really worked, but I've got to say that I never really did anything else to stem the weight-loss, also stem the weight gain in my 20s if I'm honest. I kind of noticed I was getting bigger but it wasn't that point, I think, yet, where it was really depressing me, or bugging me.” “We probably had two years of eating, drinking, and just enjoying the first couple years of our relationship. That's probably where I got to my heaviest. I think I got shy of just about 20 stone.” “I wasn't really doing any kind of physical activity. If I ran out and grabbed something from Tesco, for instance, for lunch it would be like a sandwich, crisps, chocolate. It was what I'd have every day over the course of the day.” “We feel like we've given everything. We worked really hard over that winter period, so when it quietens down, we make the most of it. That's our unspoken rule at work. That's why we get away with going to the pub at lunchtime and stuff like that because everyone knows how hard we worked previously. You felt like you'd earned it.” “I think it's just the laziness. It's the doing nothing piece, that's what makes me eat. When I've got nothing to do, I'll eat as something to do.” (P6) “I actually know exactly what it was, but basically I started a job and I was working 14, 15 hour days in an office sat down. You'd be out drinking every single day with clients or with colleagues. It just basically meant that I was taking in a lot more than I ever had done previously. It was just no exercise that was going with it because I was always either working or out.” “I had various attempts. I'd go back to the gym. I didn't do it consistently. I just did it every now and then, but my biggest problem was I think at that point was the fact that every evening after work, we'd always go for drinks or we'd go out for dinner with a client, so I was pretty much out eating restaurant food or drinking in pubs pretty much five or six days a week.” “My actual physical health and my mental health were just all over the place” (P1) “I was at work and I'd take some buns because the bakers was just down the road and the apprentice that I had, he used to go down the road and I'll say bring me a couple of cakes and a bun and this, that and the other, which I would eat as well as my sandwiches. I was eating badly as well and I wasn't thinking at the time.” “I was drinking a lot. I used to go to the pub lunchtimes and have two or three beers plus what I could eat and all the wrong things I was doing” “I think really as I got older I got lazy in exercise and that. I do drink a lot. I have to admit that.” (P19) “Then you kind of get out the swing of it, and then you just think, "Oh, it's raining. Oh, I can't be bothered." “Or, "It's cold." All that, just to snuggle up in bed. Then you get into the bad habit and you lose your self-motivation and you just can't even get out, then you stop going.” (P17) “Whether or not it was coming up to Christmas, my birthday, or a wedding, a christening, a nice do. It was always for something that was coming up, and then after that, you sort of thought-- You've done what you needed to do if you see what I mean?” “I suppose it's because then, you don't have something to aim for, you kind of fall back into old habits.” (P8) “I was busy looking after other people at that time, so I didn't think about myself too much.” “Rather than I suppose really try and do something about it, I felt I didn't have time. I was too busy with the children, and then, I was looking after grandchildren, and then it was mum, and it was just-- I really put myself on the back burner. I put everything and everybody else first.” (P18) “[…] you start slipping into little habits and that. Then I think the weight slightly started creeping on.” “Probably again, where I've not worked, having the two children, I've dropped back in maybe the exercise, and obviously I'm not on my feet as much as well.” “Again, I've never been one for breakfast and lunch, not at the time. Then you'd eat a big dinner because by evening time, you're starving, right?” “You had a stressful day at work, you sip on a glass of wine and that glass of wine turned into a bottle. It's just all calories that you don't really see it because you think one drink and at the time, you're oblivious to it all.” “I think that as well. I don't know, there's always tomorrow. You tell yourself, "I'll start that next week," blah, blah, blah. You never do” “Then I thought, "This is really good. I can do this," but then again, it's too tempting. There's Christmas coming up, or there's Easter. It's someone's birthday, and then it's one of [inaudible 00:13:07], we'll go for a barbeque, we'll have a couple of drinks. Once you make that bad hiccup, you fall off the wagon, you're either that type of person that can dust yourself off and jump back on, or you go, "Uh-oh, I'm stuck. I need a helping hand." (P13) “I didn't really use to eat properly because I didn't always have time.” “We used to eat too much, because I think the recipe said it was for four people. Willy would say, "God, that's not enough," and we would do it for eight, and we would eat that between the four of us. That kind of thing.” (P16) “I've probably not even ever had the willpower to do anything before. I don't know.” “I've never really paid attention. I never really used to weigh myself.” (P20) “I'd probably say I was your typical yoyo dieter. Able to withhold it if I have the inclination to do so but could quite easily slip back into bad habits.” “It's very easy to try to pick up those eating habits. / Then kind of busy times at work, you end up eating rubbish and you get to the end of a week and think, "Do you know? I've had three takeaways across the week, I've done this” (P2) “Every night then became a social event that we would eat and we would drink and very quickly the weight started going back on. I was probably up to about 16, 17 stone within a year of us being together.” “Put weight on, then we moved in together” “When we first moved in together, I was working in a different city, so I didn't see very much of my husband and it was a case of, I would get in, he would cook me dinner, I would eat my dinner, I would then go to bed because I was up at five in the morning. Then we started living quite separate lives and the only thing we'd do together was eat together in the evenings. The following year, I was up to about 18 stone” “have a very bad relationship with food. Food cheers me up” (P1) “If I'd start eating sweet things- if I bought five donuts, I could eat all five in a day. In my house, there has to be no biscuits, no crisps, no nothing. You just hope that [inaudible 00:05:27] got visitors, because I couldn't do sensible snacking. Then I was workings at a job I didn't particularly like. I had split up with that boyfriend, and I'd go at lunchtime get my sandwiches and then I'd get two apple cream tarts and sit and eat them at my desk. [laughs] It just- Then I'd probably have few glasses of wine in the evening.” “I'd lost all my drive.“ (P5) “I think we had a more sedentary lifestyle then and so we both ate a little more, motioned a little less” “The old fasting thing didn't work” “[…] I got more sedentary, which was unusual for me. That regular work hours and I was about to have a family, and we had friends and we were invited and we invited back at dinner parties in the evening or lunch parties during the day, and I started eating more than ever had done before.” “I had a wife who was a really good cook.” “There're had impressive bouts of training, I would train excessively, say, for a month or so. Then I would stop, because I find it boring and annoying” (P10) “The problem was my day job was fine, but then I had very frequent 12-hour shifts” “What I started doing, at nights, when I had to stay awake, like when I had consecutive three nights or four nights when I had to stay awake, I noticed that I could stay awake if I eat something.” “Then, when I came back, again I started my on-calls, exams and although I could see that I was putting on weight, I just didn't do anything.” “I was justifying it in my head that I need to eat protein.” “I think if I look back, if I reflect, this is the first time I am prioritizing my weight. This is the first time. Otherwise, I haven't thought of it. Either work or something else came up, so my weight was always the bottom of my list.” (P21) “[..] for some reason, I really put on weight. I enjoyed the lunch with all my colleagues and comrades and, again, this time workload increased a lot and I had a lot of problems as well, because of the massive workload I had at the hospital. Then I had problems also with my family” “I did not eat very considerate. I just ate because I like to eat. I'm obviously very fond of sweets as well. This is one of the main problems. I think I always thought if I have some chocolate or that makes me stronger or I can survive this situation again also (P9) “To be honest, I've been really, really busy, and there has been priorities all the time. Medical school is a nightmare, as you can imagine. It's just study, study, and study, and lots of exams. Then after we finished medical school I got married. I moved here and had to go through more exams. Then I got my license and I had to go through more exams to specialize, [laughs] and I have kids. It's just that I always knew that I have to do something with my weight. It was probably my laziness or priority really, I could have gone to someone for help, which I didn't do.” “Even though I've always been overweight, I don't think it could ever knock my confidence off really. I probably masked it with my intellectual ability.“ (P12) “There wasn't a regular pattern. It was just as and when really.” “It's not really something I thought about.” I don't care. I'm going out. I'm still going out and getting drunk. I'm not thinking about calories.“ (P4) “I'm aware but I cannot do anything because I think I need more encouragement. I cannot go to the gym. I was so busy at work. I'm working Monday to Saturday and I only have one day off during the week. I've got so many excuses. “You're on your feet for 12 hours. I get out of the house at 6:30 in the morning. I go home at 7:30 to eight o'clock PM. So you're on your feet the whole day. I know it's not an excuse for exercise. You wanted to do something about it but your body cannot do it anymore.” (P14) “I found that my habits got quite bad again. I'd probably say I was your typical yoyo dieter. Able to withhold it if I have the inclination to do so but could quite easily slip back into bad habits.” (P2) “When I met [unintelligible 00:09:24] I basically stopped. We probably had two years of eating, drinking, and just enjoying the first couple years of our relationship.” “I guess because of that, it takes a lot for me to start playing something or to get back into it because I can't get confidence that I can do it without it actually dislocating.” “Stopped because I'd met my wife and then I was due to go to overseas for three months for work. This was 2016. I probably had my last session in August 2016. Then met my wife and we were going to overseas in the week and stuff, so I was canceling the appointments to go on [chuckles] dates.” (P6) |
| **Personal Characteristics** | Diverse personalities | “I kept my circle very small. I get a bit nervous now meeting new people. If they're quiet or if I get a sense that, "Oh, they don't like me," then I can't stay more quiet and I prefer my own company. If you get a good vibe from people, I'm just me. I'm chatty. People tell me to be quiet most of the time. I talk too much.” “I'm pretty laid back and easy-going but I am anxious in the sense of not scared to go out and things like that because I know there's different types of anxiety. My anxiety is overthinking things, worrying about things that haven't even happened yet.” (P13) “I'm probably quite an extrovert. I say and do what I think, get corrected later, which is not always a good thing, but I think that's also helped in making a choice and sort of having a little chat with myself to say, "Oi, sort yourself out." Yes, probably more of an extrovert person.” “Easy-going.” “I suppose it depends on what I'm doing. I think if I'm comfortable. If I need a push I would need someone else to push me, but I can also pick myself up to get on with stuff if I need to.” (P2) “I'm an extrovert. / I've done most of my MBTI testing and all sorts of personality tests and I always come out as the extrovert. Not as much as I used to be but I guess in terms of where I draw my energy from. / I'm more easy-going.” “If it's like I said an external project, I can be very self-driven, very self-motivated. I think where it's something about I am not left outside of my comfort zone, then I definitely will rely on other people.” (P6) “I would say I'm more introvert.” “I like parties, but I feel exhausted just after a while. I feel like if I could go home and just relax on my couch and watch Netflix or something.” “I'd say more anxious.” “I'm more externally motivated.” (P21) “No, probably from being with people.” “Easygo-- well, probably a bit of a mix of both now. / I'm probably more anxious in life situations. I think that's more like, "Oh, what if something bad's going to happen?" I think because I've had quite a lot of negativity in my life.” “No, external. Yes, I'm not so self-motivated. I need to be pushed.” (P20) “Others would probably say I was an extrovert” “I'm kind of a bit of both.” “More easy-going.” “I would always have said that I was quite self-motivated, but then when I was ill, I think that I really struggled to motivate myself to do anything. I'd say I am more self-motivated than not, but I would say that I definitely am prone to pangs of lacking motivation sometimes.” (P1) “I like both, if I’m honest with you. I have moods where sometimes I want to be with everyone, but then sometimes I want to be on my own, I want to do stuff by myself.” “No, no, I'm easy-going. I'm easy-going.” “I am self-motivated in some places, but I do need a little push now and again. My wife would always push me to say, “Look, you need to do this or that” (P4) “I get energy from being with people. I like to be with people but then there is times when I do like a bit of peace and quiet and be on my own” “I rely more on a lot of other people for motivation for this kind of thing, if I'm truthful.” “I'm easy going. I'm quite easy going but I can be a very anxious person. I am a person that likes to know what's happening.” (P16) “I am a very weird person.” “Yes, because I can be the two extremes. Usually, I am quite introverted, I'm very shy. I find it difficult making friends and that kind of stuff.” “I'm with people I know, who I'm comfortable with, then you can't shut me up.” “I'm easy-going. I go with the flow. I can lead, but I'm quite happy just going where the will and the wind takes me.” “I guess, depends on what it is, what I'm doing and what I'm trying to achieve. I can be very motivated and get things done, and then, other times, I can be very laid back and think, "No, that can be done tomorrow."” (P17) “An extrovert.” “I love social socializing number one. If I have time, I would socialize every day. I love talking to friends, to my colleagues. It's good for your well-being. Social life.” “I am actually easy-going, but I'm anxious if I'm with my chief executive, for example.” “But I'm easy going. I can go anywhere with anyone.” “Oh no, I'm self-motivated.” “I can see people I'm inspired of what they wear, what they look, and now how come you got 3 inches waist.” (P14) “You see, there are many times where I can be very reserved, I can be very quiet, I'd be focused, I'd be sitting back and listening to what everybody's saying. Then there'll be the exact opposite, where I'm the first one out, I want to get my point across whether that's in the office, whether that's work. You know what I mean? They'll be times when I'm really loud or among the loudest in the room. It isn't a pattern as such.” “I think that's probably changed in recent times. I would probably say I'm more anxious nowadays. I don't think I've been easy going since I turned 40.” “Well, generally, I'm more self-motivated in respects to the weight-loss side of it as well.” (P3) “I've an all or nothing personality.” “I'm always really outgoing and bubbly. I probably always put a face on with that.” “I'm pretty easy-going. I've just had some anxious times, but I'm pretty easy-going otherwise.” “Yes, I'm not-- Well, I'd give myself projects. Like I'll paint all the garden fences this weekend or something or I'll decorate a room. But when it comes to going to the gym, no. When I went to running club, and my friends would call me up and say, "See you down running club. We'll go and--" That would definitely cheer me to go to the class, because I'd want to see my friends.” (P5) “I'm quite an impatient person I need something to be easy to follow.” “I think I'm a bit of both. I come across as very confident but I'm not. I'm really not. I'm a really, really insecure-- I'm very insecure. I get very ashamed of myself very quickly. I put a lot of pressure on myself.” “Anxious.” “I'm self-motivated. I have to be self-motivated to take on external motivation.” (P1) “I would say I'm more of an extrovert.” “I am definitely a people's person.” “I'd say I'm more easy-going.” “I suppose I'm not by nature, I'm not a very anxious person and I just take things in my stride.” (P11) “Initially, I could come across as being introverted, but I don't think generally I am once I get to know people. It's also, possibly, to do with me as well. Once I'm comfortable with people, I could be quite extroverted.” “Quite an easy-going person.” “Certain things, I think I need to be pushed. Generally, I'm not the sort of person that needs to go to the gym with someone.” (P8) “I like being with people but I also like to be on my own as such but I sometimes like to be able to just leave a night on our own. Just the two of us to go out or be at home on our own but I also do like to be with people, friends, family get together to do things.” “I'm easy going.” ”Self-motivated. If I want something done I do it myself. I look at anything, "Oh, I need that, I need to do that" and I get up and do it.” (P19) “I'm a people person.” “I suppose I'm pretty easy-going, but I do get anxious about silly things, I think, on occasions. I try not to” “If it's something I want to do and I can do it, I will do it.” (P18) “I'm very much extrovert.” “I am easy-going.” “No, I am a self-motivated person, and I do a lot of thing really.” (P12) |
|  | Inconsistent perception of overweight | “I went down the pan a bit. I hated myself. I felt fat and then you start thinking about all your past and everything. You just fill yourself with self-hate really” “I think my clothes as well didn't do me a favour because most times if you wear jeans and that they're fitted, so you'll notice if they are tight, but when you're wearing leggings, I call them grow bags, they grow with your body.” (P13) “I've always been very, very skinny. Even when I started putting on weight, I put on weight, I started but the rest of me still had skinny arms and skinny legs.” (P6) “I started gaining weight even before that, but it wasn't that much of a problem for me because my clothes were still fitting, but about a year ago, I first noticed that I need to change the size because my regular clothes are not fitting me anymore.” “That wasn't that much of a problem because I would say I was quite curvy at that time, so even though I gained weight, the weight was quite proportionate. I wasn't that much worried.” (P21) “I've always been bigger. I've always been a bit bigger.” “Yes, possibly, but it’s hard to tell, because I've always been bigger.” “I wouldn't necessarily say, because I always see myself as big anyway.” (P20) “I was always on the slightly chubbier side, but never really massively. It was just that compared to everybody else, I was a bit bigger, so yes, I guess it balanced itself out. I imagine I probably got taller and it probably didn't look so bad.” “Interestingly, I always thought that I'm still overweight. It's only when I look back now and I see some of the pictures of me then. I just think, "Bloody hell, if only I'd known, I would have [unintelligible 00:07:06] and stayed the same weight.” (P1) “It never hindered me. Obviously when you're young, you don't really think about it, to be honest. / I went into college and really, I think, it didn't really affect me then but obviously I started to become more self-conscious about myself then about being overweight because you see a diverse diversity of people. / Since after eight when I became overweight, I was never happy with my weight.” (P4) “I've always been a big lady. / I said, "Not to worry, it's just I've always been big." / Oh no, I've always been what they would class as obese. I've always been a big girl. Always been in clothes bigger than my age group. I've always been. Always been big” “To be truthful, I've never really been happy with my weight.” (P16) “You feel insecure when you go out with your friends because they can wear sleeveless, they can wear backless. When you go out to the beach they wear two piece. I only have swim suits. Then you cover yourself, so now you're more aware of your body image.” (P14) “I knew I was obese, not just by the calculations, I could feel it, I could see it, if that makes sense.” (P3) “I think that's been the bit that-- I've always-- I have never been a stick-thin child or adult, even after having my two children but I was always like about 12st, 12st 7-ish, and I was happy with what I was. We always want to lose a bit of weight, don't we?” (P8) “Yes, I was always quite big anyway, I was never a skinny person.” “I didn't worry about it though, Marie, but I didn't-- I suppose I couldn't do as much as I used to, but it didn't worry me. I didn't feel ill. I felt okay. I didn't have problems. It didn't worry me at all. I carried on as I was.” (P19) “I couldn't lose it and I suppose from then I just accepted, "Oh well, that's it. I'm this weight now," and that was it. I just carried on.” (P18) “It looked ugly, persons who don't look ugly immediately when they put on weight, but I was one of those who just got a round waist and I started looking ugly and I didn't like that.” (P10) |
| **Successful Weight-loss Attempts** | Various dietary approaches | “Started to really become more conscious of what I was eating. Started exercising more and I lost over two and a half stone. Things were moving in the right direction.” “I think it was more she portion-controlled in terms of the size of the dishes that I was having because what we ate wasn't bad food, but I just liked a lot of it, I think.” “I think it was probably a bit of both. Made sure that I had smaller portions and then the fat that comes naturally then by [unintelligible 00:05:36] it was I feel like a bit of a blend. “…] I just survived off of black coffee, Lucozade and Marlboro Light cigarettes. That was basically my diet at Uni.” “I was also more conscious of the fact that I was exercising so I should probably eat slightly better, so I probably had a better diet.” “I cut out a lot of things like heavy carb. I would have three meals a day rather than five different meals.” “I cut out all fast food, party weekends. I still went out to restaurants but I was conscious of what I picked at restaurants. I stopped drinking a pint of cider a night. I moved to gin and slimline tonics. I lost almost three stones.” (P7) “There was no alcohol. We [unintelligible 00:26:37] what we were eating, I also had my operation on my nose so I was at home, and I wasn't really eating much. By March, April, I was able to lose about two stone.” (P6) “For the first month, I told my aunty from Monday to Friday to eat pulses and rice, like that. Then enjoy yourself for the weekend.” “I was drinking a bottle of water every day.” (P15) “You lose weight when I was working night shift. Because I cannot sleep during the day, after night shift and then you go for night shift without even had sleep. I haven't even slept. It reduced my weight” (P14) ““I ate less, yes, but I wouldn't say I was counting calories” “I did lose weight. I mean, I'm still fat” (P4) “It was just a diet at home to make sure I didn't drink as much.” “They watched what I ate.” “I probably ate less, but I also ate more of the right things and take some [unintelligible 00:03:28] at work” (P19) “Before that, I had tried to do Slimming World on two occasions.” “I enjoyed it. I think I did it first off, I'd lost 22.5 pounds in about seven or eight weeks.” (P13) “My biological mom and I did go on some diets when I was older, when I was a teenager. We did join a diet group and we would exercise once a week. I did lose quite a bit on that.“ (P16) “I've got ADHD, so I take [unintelligible 00:04:55], and, obviously, that suppresses your appetite, because I think it's actually got speed in it.” “I did lose a big chunk of weight, I lost five stone in total, but that was probably over a couple of year period, and I don't even know. My eating habits wasn't good. I technically didn't eat anything. I would just drink Red Bull.” (P20) “[…] it became very easy for me to stop eating altogether, and that's almost what I did, was I would not have breakfast, I would not have lunch. I would then go to the leisure center, and so at dinner time I was at work, and so then my friend would say, "Oh, do you want something to eat?" I'd be like, "No, no, no, I'll eat when I get home." Then when I got home, mom would be like, "Do you want some dinner?" "No, I ate at work." Quite very easy to stop eating, and I started doing exercise classes.” “Well, I then started diet pills to get myself a bit of confidence and I got myself back down to about nine stone.” “They were from a place where I used to gamble. So probably not certified.” “No, just didn't make me feel hungry. I did that for a little while and in-between I've done the [inaudible 00:24:23] Probably about a year and then I started to feel unwell from talking it” “I was down to about nine stone.” “So then we did Atkins and then I got down to about 12.5 stone when we got married.” “I did Weight Watchers, I'd done the Cambridge before, I did the cabbage soup diet, I did crash diets, like the three-day crash diet, it’s like beetroot, and cottage cheese and its horrible.” “So I started Slimming World and I threw myself into it” “I'd lost quite a lot of weight in 2010 because I had pancreatitis, I had a gallstone and I lost about four stone in about four months.” ”I was so good when I was pregnant, so, so good, I only put on one kilo through my whole pregnancy, and I lost weight after having him, just from having him. I used to eat really healthily.” (P1) “I used diet pills then. I went up to size 16 and I lost the weight that time really successfully” “Then I tried slimming pills” “That was really easy. I was like, "Wow. You just never want to eat." That was easy to make a sensible chose for the salad, because you just weren't hungry” “Then, I went back down to a good size 10, which I was happy at that weight, so probably at that time, would have been when I was probably about 9.5 stone. That was fine, and I kept that weight off for about five years.” “[…] I did lose weight again, by going on the slimming pills the second time. But through the breakup I lost the weight, because I was really upset on that particular occasion. Every break up in my 20s and my early 30s, I lost weight. I want back to the slimming clinic and I took the pills religiously, for about 2.5 months and got down to 10.5 stone, because we were still drinking, but I just wasn't eating and I was drinking that Redbull stuff” “I tried to follow a Keto meal plan, and with key times. So it was low carbohydrate and then there is meals there, but again, it's very much a lot of the same foods. I lost a stone and a half” (P5) “I then started fasting at the age of 34 or something like that. I took ephedra containing [unintelligible 00:01:49] that kept my hunger down” “I started fasting and doing sports.” “All I did was skipping meals and eating mostly egg white, because I had read that egg whites is burning fat and it seemed to work great with me, because I then you should promptly lost weight. In those days, I had no difficulties in losing weight and my skin was contracting again very quickly and things turned out fine. The other thing was while I was losing weight, my mood went up and became fast. It was sometimes a bit like a drug. I was more active, I was more alert and I quite liked that. Then I had a time when I didn't have to fast, because I was so busy and so active that I had so little time to eat during the day that I maintain my ideal weight.” (P10) “At the same time, my eating habit changed a lot, hugely, because I was getting food from the hostel diner, and it wasn't tasty at all. Not at all, in comparison to my mom's.” “I didn't know how to cook, so I was mostly having rice and lentil soup with a little bit of pickle and sometimes a small piece of chicken. It's not that I had that food just to lose weight, it's just that that was the only thing I felt that was tasty enough to eat.” “When I got back home after a few months, two or three months, my parents would always say, "You lost a lot of weight” […] I actively started dieting, I had lots of salad.” “My mom made different kind of salads and I just had the salads.” “At that time, we started using Noom and we got used to it very, very well. Both of us, we lost weight” “No physical activity” “I just counted my calories, that's it “ (P21) “[…] I think I was doing a lot of sports and I tried to eat less, I must say. That worked quite well and I was a little bit distracted, because I had to study so hard. Then I got distracted and I didn't think so much of eating. As I was out so often in the library of the medical university, I wasn't always thinking of eating.” (P9) “After my secondary school exam, before I started university I managed to lose weight cautiously. I was watching what I was eating and maybe I was walking more. That's when I managed to lose some weight.” (P12) “My biological mom and I did go on some diets when I was older, when I was a teenager. We did join a diet group and we would exercise once a week. I did lose quite a bit on that.“ (P16) |
|  | Increased physical activity | “Started exercising more and I lost over two and a half stone. Things were moving in the right direction.” “I started going to the gym when I was probably around 25 and that was really quite good actually. 25, 26 and that was quite good because I got into the habit of going.” “I got a personal trainer, so whatever he told me to.” “I would go to the gym five times a week.” (P1) “A friend of mine was a personal trainer. I went to see him twice a week after work. That was around the age of 30-31. I did that for a year. I was basically going once or twice a week to see him. I definitely never really enjoyed it, but I did start to see differences “ “So I was like, "I've got to lose weight," so the first start of last year, I think the first three months, I'd lost like an inch.” (P6) “I did so much walking like I haven't done for the last five years with my uncle, he liked walking.” “Then what I decided to do as well in the evening he had like one of those cycling machines and I did a bit of cycling every other day” “and I got my weight down” (P15) “I did join a gym, and then that was going quite well. I did lose some weight, and I put some of the muscle back on.” “I went for a good year.” “I went on average about three times a week.” “Probably lost about a stone, I think.” (P17) “[…] I started doing exercise classes. Then I would then after I'd finished work at nine, I'd then do a spin class 9 till 10, and then go home.” “I would do circuit training, I would do step aerobics, I would do spinning, you name it, I would do it. I would have a swim, I would do one circuit class, and then they used to do back-to-back circuit classes, on a Friday night, I'd do two back-to-back classes. Then I started exercising, and very quickly weight started to fall off of me” (P1) “[…] they started doing yoga and different physical exercise.” “I would say it was just maybe 5, 10 minutes every morning or every evening.” “ […] I walked a lot. If I had to go for shopping, rather than taking a car or something, I would just walk. If I had to go to the tailor or anywhere-- Maybe every day I walked more than an hour.” (P21) “[…] I think I was doing a lot of sports and I tried to eat less.” (P9) ) “Just eat, go to the gym. I thought if I'm going to the gym, going swimming, I'll start to lose weight.” (P4) “We did join a diet group and we would exercise once a week. I did lose quite a bit on that.“ (P16) |
| **Motivators** | Health  Family  Self-confidence | “"[…] I'm 40 now. I need to lose weight." Obviously, I've got kids. I've got young kids. I don't want to have a problem later on in life. Obviously, when I had kids, I started to become-- not self-conscious, but obviously I started thinking differently. It's when I was coming up to 40, I was like, "No, I need to make a difference because if I don't, then obviously my kids will suffer. If I don't get to see my kids grow up, then what's the point?" That sort of thing. I said, "I need to start losing weight," but it just was not shifting.” “It was health, not to look better. I've got a couple of t-shirts I'd like to wear because I think they look nice” (P4) “I wanted to look better because I love wearing dresses, I love going out. I'm presenting in front of so many people now. I have to speak in public, so I have to be presentable. If you're a good example to your staff and to patients, they will believe you. We have so many patients with diabetes and then how can you teach them if you're the one who is not? I decided for myself and you have a history of diabetes in the family, high blood pressure. I think I should really take care of myself now. Plus, you're not getting any younger. You have to limit what you're eating. You have to be careful because everything will be suffered in the end.” It did trigger me to look after myself and I have a personal issue with my husband. When I had that issue with him, I said to myself, well, maybe he did that because I was like this. I tried to blame myself, not to take care of myself, being presentable. It's not right to blame yourself but I think it helps to motivate myself.” “I think shortness of breath because I could not even go to the stairs that far. It was hard.” “It's hard for me to walk. It's hard even for me to sit down on the couch in my chair because you've got a big tummy, abdominal wall and it really push your diaphragm [laughs] and then I snore big time [chuckles” “Sometimes I have this reflux of the fatty foods that you eat.” (P14) “[…] my father had been taken poorly a few years ago with cancer treatment and prostate cancer, that then made me start thinking of my own health. I turned into my 40s and then, yes, it seems like a switch just then goes on, doesn't it?” “You want to be around to become a grandparent, not that I want that any time soon, but” “I was also quite mindful that in that room, and this might sound pretty horrible, but in that room, you could almost see the future. There were people there who were older than me, who probably were only just a few years older but they looked 10, 15 years older because of the way their diet-- You know what I mean?” “[…] you can see a little insight into this will happen, I will end up struggling to walk. I will end up struggling to breathe. I'm asthmatic already. You know what I mean? It's all that type of thing that you then see, and that was a good point” “Family certainly are without a shadow of a doubt, and not just my two, because they're the sort of older children in our family. I have nephews and nieces who are brand new if that makes sense. Some of them are still in nappies and knowing me as uncle Participant, I want to be their uncle Participant for as long as I possibly can.” (P3) “[..] it's I guess basically a last chance saloon as well because I realized that my health wasn't-- I wouldn't say started deteriorating, but I started to get health issues as well, like breathing. One day I woke up early and said, "Look. I might as go into work early," it was just like six o'clock in the morning, but for some reason, I was breathing real fast-and I thought, "What the heck is going on?” “Then he said that I might have COPD, but then he said it might be, I have too much weight pushing my lungs up or stuff like that.” “The other thing is my walking because I had that breathing problem. I'm slow in walking. I was just slobbing around.” (P15) “Originally, it was really around wanting to lose weight and stretching my knee” “The two main motivators, the biggest one was obviously the wedding, without a doubt, that was the biggest one. The second one was also I guess the health reasons to do with my sleep apnoea. I don't want to have to sleep with the CPAC machine for the rest of my life. They said that the biggest thing that you could do that helps is to lose weight.” “I don't want to keep having the psychological nightmare stuff like my knee dislocating and stuff like that. I want to be able to, whilst I'm still young enough to be able to do that because I'm starting to think forward. I've started to have these fears of being 40, 50 and basically just not being able to do physical activity, because I just didn't get my body in shape to do it when I was younger.” “I think things like having kids and we're talking about having a family and stuff. I guess it's one of those things that's starting to play in mind now. I want to be able to run around and just generally do that type of physical stuff with your kids and stuff, which at the moment I don't think I can do” “I just think this time now, I've got a point now where I want to turn it around. In a weird way, I always feel like it's the last chance saloon a little bit, like I'm running out of time before I hit the, you can't come back from this place.” (P6) “I think, in terms of physical health, I think it would be a lot better because of the fact that I am often short of breath and I think that part of that is extra weight which I'm carrying and my knees that are constantly [unintelligible 00:37:00] We go for long walks and I end up with sore knees. I've always had bad knees but I think it definitely doesn't help with my size.” (P1) “I did want to improve because I started to put on quite a bit of weight since I'd retired.” “You don't have the extra weight. I do feel so much better.” (P19) “Because of the weight that I put on and it was such shock, I thought, "No, I need to do something. If I don't, I'm a heart attack and stroke waiting to happen."” “I want to live for a good long time. I want to be able to move and see my grandkids when I have any, hopefully, and be able to get down on the floor and play with them and do all that kind of stuff. With how I am, I'm going to end up living down, down in the entrance hall of our flat because I won't be able to get upstairs” “I thought, "I need to do this for myself and my own self-esteem and for my family." (P17) ”I was conscious of weight gain because my knee. Yes, I've always been careful because of that problem but now as I've older, all my joints hurt as well” “After seeing that, I really felt like if I could reverse my diabetes and obviously lose weight with it. that was really the two things that I wanted to do and inspired me about it all” “What I'm hoping is if I can keep it at bay as long as possible as a result of doing this, that's really what I'm hoping for” (P8) “It was just the diabetes I was interested in.” “Health-wise I think because I realized it's not good to carry extra weight, it just slows you up and other things. My hip was getting more painful and things” (P18) “I obviously needed to lose the weight for my - what's the word? - Self-confidence and things like that, but also physically. I needed to be fit and healthy for my children. I want to be able to run around the park with my kids and not be out of breath. I want to be the fun mum.” “Also for health. I've got arthritis in my knee, I've always got back pain. Whether that's to do with care work, probably my weight as well. In fact, it probably is more my weight. All the aches and pains, the headaches, the diabetes. I need to be, what's the word? I need to be on the game for my children. I don't even know what the word is. I need to be on form. That's it. I'm top form for my kids. I don't want to be in a wheelchair in like 10 years’ time because I'm crippled with a bad back and dodgy knees and stuff. Having a young baby and a toddler, I need to be fit and healthy to keep up with them.” (P13) “[…] it was a bit of an awakening call for me because at this age, my biological dad did have a heart attack. I'll be honest, I was feeling a bit that my body was being a bit strained.” “[…] when he said to me, "I'm getting worried about you. You're piling on the pounds," then I did get worried. I thought, "Well, maybe and I really need to do something about this really” “At first it was weight-loss and to look good, to feel healthier.” “[…] be healthier and be there for my children when they get older and stuff like that and they won't have to worry about me having any or small amount of ill health.” (P16) “I know now what it's like to feel lighter, to feel better. Before, I never knew what that felt like, because I've always been big.” “Yes, it is, because obviously, when you're smaller, you feel better.” “Even colleagues at work were like, "My God, you're so happy." I was on a high. It was like I had a spring in my step and stuff, maybe more energy about me.” (P20) “It was seeing pictures of myself and then realizing that I wasn't very happy with myself and my body image” “We want to start a family it's obviously impeding on that," “[…] I just have very sore knees, like first thing in the morning my knees are quite sore which again, has been quite a wake-up call and it's something that you don't want in your 30s.” “[…] clothing as well, and personal image.” (P2) “I want my confidence back and I know that when you don't feel good about yourself-- There's nothing that makes you feel better than when you put something on and you look nice in it.” “Definitely looks and how I feel about myself, so it's for myself to feel better, but also about being breathless a lot. Going up the stairs specifically. If I was on the phone my mum would go, "What on earth are you doing?" I'd say, "I've just walked up the stairs." I'd be so breathless.” “Rolls of fat, is just disgusting. It's just that looking-- I wouldn't have a-- I couldn't see my feet at one point” “But it's just all about my whole well-being as well. It's all interlinked, isn't it? Our health, feeling good about ourselves, and being mobile. I don't want to be someone that can't get off the sofa in a few years’ time " (P5) “The first motivation when I was young was that I had a feeling that getting fat was unhealthy and it would slow me down, which I couldn't afford, because I was really very busy and doing a lot of work. Then, when my children were born, I didn't want to be a fat dad, so I decided I would like to look all right. Then, when I was past the age of 60, I got a heart condition and I put on two things” “I felt a bit panicky, because I knew if I didn't lose weight, I didn't know, I might die.” (P10) “It was like a mixture of, "I want to get more energy and I want to fit into my clothes” “They think it's just PCOS because my testosterone was quite high, also. That's another reason that I think I have extreme hair fall now and I started having facial hairs a lot more than before. For me, that's also a driving force now. I want to get rid of all these problems.” “From December, January, I stopped going to parties over here also because I knew everyone is going to comment on my weight, number one. Number two, none of my clothes fit, the party clothes, and I didn't want to buy anything new. That was another reason. Fitting into my clothes, that's the huge agenda in my life, yes.” (P21) “Now, I have the feeling I am taking control of my life again, which is really I always wanted to do.” (P9) “I'm more cautious about my health now. Now that I am getting older and I think about my kids, I need to live longer and stay healthy for my kids for longer.” “Obviously, I do understand that people look better when they are slimmer [laughs] and that's an added point. Obviously, I'm hearing more from friends, "You've lost so much weight." That's a motivation.” (P12) |
| **Expectations and Goals** | Weight-loss  Improved health  Better self-image | “Obviously when I started this, the whole point of this was to reverse diabetes, which would be fantastic if I don't have it. Great. Obviously, the plus point, losing weight is good. I feel good.” “[…] if I'm in a shop and people say, “Oh, you're shrinking, or you've lost a lot of weight,” which a lot of people have said to me, so it makes me feel good as well. Like, “Oh yes. Okay, people have noticed.” (P4) “What I want is just to be healthy. Maybe size 10, that's it.” (P14) “I know if I nail one, I nail all three [T2D, hypertension, hypercholesterolemia]” “That would be probably my main aim, my most crucial aim to make sure I could maintain that and control it.” “I would like to get back to that happy weight which was back to when I was 25.” (P3) “Get a little bit fit. Walk a bit more fast.” “If I can walk a little bit faster and breathe a bit more. Once I get my fitness to a reasonable level, take my kids to the park and all that I can maintain the pace like that” (P15) “Yes, for me actually it's not just about weight but actually, if I could build muscle and I weighed more that wouldn't bother me. It's more about how I look and feel.” (P6) “I'd just like to be within the [healthy BMI range] [unintelligible 00:36:41]” “The second point in terms of how I feel, I think it will feel fantastic to be back to where I was to be able to put on.” (P1) “I want to be able to move and see my grandkids when I have any, hopefully, and be able to get down on the floor and play with them and do all that kind of stuff.” (P17) “At the beginning? Number one, was my diabetes. Then after that, I thought, "Well, if I could lose some weight, I know that will be good." That was my other one to feel better about myself” “I can wear nice things, and I feel attractive in it.” (P18) “I just want to feel healthy. I don't want to be trying to get up off the floor, taking me 10 minutes and knees hurt and stuff. I just want to get to that, feel good. I want the doctor to say to me, "Look, you're not at high risk anymore for getting diabetes” “To be told that they are no longer an issue and to be happy and be the weight that I want to be that will mean absolute everything to me.” (P13) “Being able to be active. Enjoy running at the park. When I go on walks, and don't get breathless, and say to my kids, "I can't do this now," or, "You do or do this with your brother," or, "You carry on with your sister. Your Dad and I are going to sit down," I don't want to be like that anymore.” (P16) “To be honest, now I'm a 16, I want to get down to a size 12 to 14.” (P11) “I just want to feel healthier and feel better, like we're saying about the bending over, the walking up the hill, the tying your shoes or painting your toes, just to feel better.” (P20) “I think I'd like to get my BMI between 26 to 28 max, and probably my weight, I'd say 12st 7 I think, is reasonable at the moment.” (P2) “To be more self-confident. To have a more-- to manage it better. To be able to maintain it better. Not to get to a target and think, "I can start eating again," in a bad way. To realize it's for life” (P1) “If I get to 10 stone which is what I weighed when I was nine months pregnant I'll be more than happy” “If I could get to 10 stone, I'd be absolutely thrilled” (P5) “I would like to get to a healthy BMI. A happy weight” (P10) “My goal is to be within the healthy BMI range.” (P12) |
| **Weight Management During COVID-19** | An opportunity for change | “I think with COVID, not much because I went on working the same as before and I live in a very, very nice place. My flat is at the seafront, I have sea view in flat. I live on the top floor. I have got a fantastic view and I'm going out every day for most of the week. Yes. There wasn't much change in my lifestyle other than I stopped shopping.” (P21) “[…] at the moment, I’ve quite enjoyed it a little bit” “Every day, I go for a walk for at least five miles. I could never have done that before but I've taken the option to actually make sure that I got to get out and about too. I've been going for walks more, I have- obviously, not going out with friends for dinner. That's been really helpful because of the fact that I thought it'd be so easy to slip into drinking every day like everybody else has” (P1) “Obviously the three weeks I took off when I decided to close the shop, I think it was the best three weeks I've had for a long time. One, because I spent time with the kids, and I haven't done that. They really enjoyed that, so for me, I'm like, “Okay, fine. I'm not making money or whatever, but the three weeks I spent with my kids, I'm never going to get that back because they enjoyed it and I enjoyed it.” We'd go for walks together. We'll talk about stuff. It's something that I wouldn't have done, so I don't really think it's like a bad thing.” (P4) “I did get a bit down at the beginning, but I did pick myself up. Even though I had been busy around home, but not enough, I felt tired, but my husband said that's because we're housebound, and he said it's perfectly normal. / I just feel that COVID-19 has brought us closer together as a family. We talk more, and stuff like that, which is good.” (P16) “I wasn't overly worried, to be honest. I followed the guidelines and sensible with washing hands and social distancing and all that kind of stuff. I think one of the vulnerable groups, as I'm asthmatic, as soon as it was mentioned about locking down and staying at home, I think my boss did it on the Monday.” “I mean, food-wise we've been quite lucky. We've been able to get our shopping delivery […] and they've been pretty good.” “We've been looking through cookbooks and bits and pieces and being inventive with whatever turns up.” (P17) “Actually, people are saying that because of COVID they tend to eat more. But if you have self-control and you have discipline and you have a target and you're focused, you don't have a problem. / I just wanted to stay focused, positive.” (P14) “I've given the isolation period and stuff quite a bit of thought as to how it's affected stuff, but it's not affected me to go and eat packets of Digestive biscuits or to go and eat all the bad stuff, you know what I mean? It's not affected me-in that respects, that's probably a better way of putting it.” (P3) “I couldn't get out of them but lockdown has done me the best favour in the world because it’s just made it so easy.” “Well, I've used the diet- I've used the COVID in my favour because it's meant that I haven't had to join in the supermarket riots.” “I've sort of used it to my advantage, the COVID, it's made it a lot easier. It's also made it easier because I haven't had people commenting on what I'm doing.” (P5) “It hasn't affected as far as I am aware. It hasn't affected my mental and emotional well-being“ “For me, whereas I knowing it's impacting a lot of people mentally, thankfully it hasn't affected me in that way, so I thank God for that. Even in terms of overeating, it has not really affected that in all honesty.” (P11) ”Now, I am very, very grateful that I was already on the program, because now we have the leisure time to continue this.” “Again, I think for us, it is really a gift that we can do this program now, I must say.” (P9) “The diet's changed the way I'm eating. How do I feel about it otherwise? It hasn't worried me. I don't feel worried about it.” (P19) “Yes, I feel, "Oh, I could just stay here and eat something", but I don't. Well, occasionally, I'll have a packet of crisps and I felt guilty. [chuckles] I think that is boredom because he's done most of that big projects, the garden's looking really good and other things, so we've had to find things to do.” (P18) “I would say easier, isn't it, because I would probably end up one or two extra meal a week” (P12) “Yes, it's been a positive experience in two senses. A, it has facilitated that it's easy to stick to your prescribed diet, because we're not being invited to dinners. We can't invite people and people are gladly accepting that. A. B, I'm doing more sports. For the first time in a long period if at all there was an earlier period like that, I have a well-regulated circadian rhythm which I haven't had before.” (P10) |
|  | A barrier to change | “It's been awful. I'm not going to lie. I've had to ring my doctor and ask for tablets just to take the edge off things.” “Again, you get all these, "Yes, I'm going to start again." You just can't. It's just trying to get yourself out of the ground. You think how long is this going to go on for, as well? You [unintelligible 00:32:11] demons. You've got a good person and a bad person. It's like which one of them's saying, "Yes, go for it. Pick them back up. Come on you can do this. By the time we're out of lockdown think of what your family are going to say. They're going to be like, 'Oh my God,' [unintelligible 00:32:23]." Or, "Oh my God, look well done for--" Then you're thinking, "Oh God, no I can't." It's like, "Look, you're having a bad time as it is, just eat what you want."” (P13) “It's been a bit of a rollercoaster since COVID has hit. I think working very long days, and with buying food at work, and not being able to access milk at the first point, coming home completely exhausted and just needing something to pick me up a little bit, I have had a lot of off days. I think in hindsight I'm very conscious of that and I'm aware that I'm not sticking to the plan as I should right now, but I'm very confident that as soon as things get back to a form of normality I will be able to snap back into it.” “I think the first few weeks when this whole pandemic hit, yes. There was an element of emotional eating because it was really worrying. Not just for me, for people close by to me and what was going to happen. Now things have become a bit more equal over that, that's how I've got back into it” (P2) “Here's the thing with lockdown. It's the emotional anxiety that I felt hasn't been to do with-- it's been caused by lockdown, but it's been attributed directly I think to our wedding. Obviously with it falling during that period, that's where the real stress and the real anxiety lay, was not so much about the fact that we're just stuck at home or in lockdown.” [Emotional eating] “Yes, it's definitely happened, yes.” “Because like I said, I'm not stressed, I'm more just bored.” (P6) “I think it's obviously the boredom and the fact that I'm at home cooking the kids or preparing 25,000 meals a day. The fact that I'm just at home is not a motivation to not eat” “Obviously, being a single parent, I feel quite isolated, because I have no one else here. I don't have anyone.” (P20) “Because that's it, even things like it's just when you're at home, my other half and I are here together, if I’m sitting there preparing dinner and I’m preparing my shake or my soup, when I’m a work it's easy because you've got so many other things to focus on. You're so busy, it's a lot easier to manage, but being at home, it's just a lot more difficult to manage, there's more boredom, there's more food around, and then I can't go to the gym to exercise, so it's just been like, "Oh, God." Every time you think, "I'm about to get a good head start on it—“ (P2) “I felt very anxious, felt very worried. I've also felt very helpless because, obviously, I've had issues going on with my mum and my dad and not being able to see them, to physically get to them. Well, to start off with, I was then in self-isolation. Then my mum was in hospital. I felt quite lonely sometimes as well, where my husband has been out for such a long period of time and just having an eight-year-old say "Mummy, mummy, mummy, mummy, mummy, mummy, mummy, mummy, mummy, look at this. Mummy, look at--" I felt quite alone that time. It makes you feel quite tired. I felt quite tired the whole way through. Quite tired.” “I've just wanted to eat constantly” (P1) “[…] I have picked a lot more than I would normally because I wouldn't normally be here in the house, I'd be out.” “There's the cooking the dinner and everything. As I've said, I've always done it. I've always cooked and it's not seemed to bother me, but there's been times when you think, "We should sit out in the garden and have dinner or something because it's nice," and I'll have dinner as well. [laughs]” “I don't think so much boredom. I think more like doing it as part of a family sort of thing.” (P8) |
